# A signature of enhanced lipid metabolism, lipid peroxidation and aldehyde stress in therapy-induced senescence

**DOI:** 10.1038/cddiscovery.2017.75

**Published:** 2017-10-30

**Authors:** Amy C Flor, Don Wolfgeher, Ding Wu, Stephen J Kron

**Affiliations:** 1 Department of Molecular Genetics and Cell Biology and Ludwig Center for Metastasis Research, The University of Chicago, Chicago, IL, USA

## Abstract

At their proliferative limit, normal cells arrest and undergo replicative senescence, displaying large cell size, flat morphology, and senescence-associated beta-galactosidase (SA-*β*-Gal) activity. Normal or tumor cells exposed to genotoxic stress undergo therapy-induced senescence (TIS), displaying a similar phenotype. Senescence is considered a DNA damage response, but cellular heterogeneity has frustrated identification of senescence-specific markers and targets. To explore the senescent cell proteome, we treated tumor cells with etoposide and enriched SA-*β*-Gal^HI^ cells by fluorescence-activated cell sorting (FACS). The enriched TIS cells were compared to proliferating or quiescent cells by label-free quantitative LC-MS/MS proteomics and systems analysis, revealing activation of multiple lipid metabolism pathways. Senescent cells accumulated lipid droplets and imported lipid tracers, while treating proliferating cells with specific lipids induced senescence. Senescent cells also displayed increased lipid aldehydes and upregulation of aldehyde detoxifying enzymes. These results place deregulation of lipid metabolism alongside genotoxic stress as factors regulating cellular senescence.

## Introduction

Cells faced with unrepairable DNA damage from eroded telomeres, collapsed replication forks or strand breaks can undergo accelerated senescence, a process of rapid cellular aging leading to persistent cell cycle arrest of otherwise proliferative cells. Similarly, conventional genotoxic chemotherapeutics and ionizing radiation can promote therapy-induced senescence (TIS) in tumor and normal cells *in vitro* as well as in both mouse models and patients.^1–3^ Onset of senescence is dependent on cell cycle stage, oxidative stress, and other factors^4,5^ so that while some treated cells may undergo TIS, others may die via apoptosis or necrosis or arrest transiently, recover and resume proliferation. The relevance of TIS to cancer treatment remains controversial, with some authors arguing for beneficial effects and others suggesting senescence contributes to resistance or recurrence.^6–10^ A challenge is that TIS remains a poorly defined cell state, lacking a distinct molecular pathway and related mechanistic markers equivalent to those that define apoptosis such as caspase activation, cytochrome C release, or DNA cleavage.

Like replicative senescence or oncogene-induced senescence, TIS displays the common pattern of proliferative arrest, characteristic flat morphology and enlarged cell size, and increased senescence-associated beta-galactosidase (SA-*β*-Gal), reflecting accumulation of GLB1,^11^ an endomembrane-localized glycolipid processing enzyme. SA-*β*-Gal is typically assayed by detecting cleavage of the chromogenic substrate *X-Gal* (5-bromo-4-chloro-3-indolyl-*N*-acetyl-*β*-d-galactopyranoside).^12^ Although the *X-Gal* assay has been used for small-molecule screening and other high-throughput approaches (for example, Bitler *et al.*,^13^), it is limited to qualitative imaging analysis of fixed cells, given the cell-impermeable, chromogenic properties of the *X-Gal* stain. Alternatively, SA-*β*-Gal can be detected with higher sensitivity and specificity with fluorescent substrates (for example, Lee *et al.*,^14^ Flor *et al.*,^15^), facilitating quantitative analysis and enrichment of viable TIS cells.

In this study, we addressed tumor cell sample heterogeneity by using fluorescence-activated cell sorting (FACS) to enrich TIS cells from a complex population of drug-treated tumor cells, facilitated by the cell-permeant, fluorescent SA-*β*-Gal probe DDAO-Galactoside (DDAOG, 9H-(1,3-dichloro-9,9-dimethylacridin-2-one-7-yl) *β*-d-galactopyranoside).^15^ FACS-enriched, etoposide-induced TIS cells were subjected to cell lysis, subcellular fractionation, proteolysis, and high-resolution liquid chromatography-tandem mass spectrometry (LC-MS/MS). Using label-free quantitative proteomics analysis, we compared the abundance of more than 2000 shared proteins in senescent, proliferating, and quiescent cells to define a senescent cell-specific proteome, which was then subjected to gene ontology (GO) and biological pathway (Kyoto Encyclopedia for Genes and Genomes (KEGG)) systems-level analysis. Complementary microarray analysis of gene expression confirmed many of the pathways detected by proteomics.

Our results revealed that upregulation of the GLB1 beta-galactosidase and resulting expression of SA-*β*-Gal is linked to a broader pattern of deregulation of glycolipid processing and aberrant lipid metabolism marked by increased uptake and accumulation of lipids. An accompanying increase in oxidative damage to lipids^16,17^ and production of reactive aldehydes^18,19^ was consistent with upregulation of aldehyde dehydrogenases (ALDH proteins),^20^ and other adaptive responses. Our results identify new pathways and candidate biomarkers linked to activation of the senescence program and suggest promising new targets to alter the formation and persistence of senescent cells.

## Results

### SA-*β*-Gal staining and fluorescence-activated cell sorting yield enriched senescent cells

**Figure 1:**
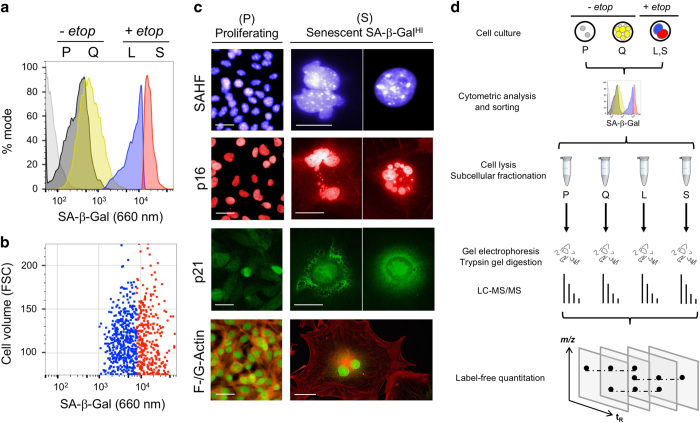
FACS of senescent cells for proteomics analysis. (**a**) Flow cytometric assay data using near-infrared fluorescent SA-*β*-Gal probe DDAOG to evaluate senescence in proliferating (P, gray), quiescent (Q, yellow) or etoposide-treated cells (L, SA-*β*-Gal^LO^, blue; S, SA-*β*-Gal^HI^, red). Etoposide-treated cells were flow cytometrically sorted based on SA-*β*-Gal intensity and subjected to downstream analysis. (**b**) Visualization of SA-*β*-Gal signal *versus* cell volume (FSC) demonstrates that SA-*β*-Gal expression is not dependent on cell size. (**c**) Imaging of proliferating and senescent cells for common senescent markers. Proliferating cells (P) growing in log phase are compared to the flow-sorted SA-*β*-Gal^HI^ (S) fraction. SAHF (senescence-associated heterochromatin foci) visualized by DAPI nuclear staining. p16 and p21, proteins commonly upregulated in senescence, visualized by immunofluorescence. F- and G-actin, known to exhibit distinct cellular localization in senescence, visualized by fluorescent actin probes. Scale bars, 10 *μ*m. (**d**) Proteomics sample preparation and analysis workflow. Cultured cells were maintained in a proliferative (P) or quiescent (Q) state or induced to senesce with etoposide. Cytometric analysis of SA-*β*-Gal distinguished senescent cells, which were sorted into SA-*β*-Gal^LO^ (L) or SA-*β*-Gal^HI^ (S) fractions. Cell lysis and subcellular fractionation was followed by gel electrophoresis and digestion via trypsin. Samples were analyzed using LC-MS/MS and data were analyzed using label-free quantitation.

As a live-cell reporter for senescence, we applied the near-infrared beta-galactosidase probe DDAOG to detect the upregulated GLB1 activity that corresponds to SA-*β*-Gal. Cleavage of DDAOG releases DDAO which is retained by cells and detected at 660 nm. When proliferating B16-F10 murine melanoma cells were treated with the topoisomerase II poison etoposide in culture for 5 days, the majority of surviving cells displayed increased SA-*β*-Gal activity, consistent with onset of TIS (Figure 1a). A similar activation of SA-*β*-Gal was induced by the topoisomerase II poison doxorubicin and topoisomerase I poison camptothecin, in both murine and human tumor cell lines (Supplementary Figure 1). Median fluorescence intensity (MFI) of SA-*β*-Gal signal in etoposide-treated B16-F10 cells (MFI=13.1×10^3^) was over 10-fold that of proliferating cells (P, MFI=3.1×10^2^) or quiescent cells (Q, MFI=5.1×10^2^). Toward obtaining highly enriched senescent cells, the 50% of etoposide-treated B16-F10 cells that displayed the highest SA-*β*-Gal fluorescence were enriched by FACS to yield a SA-*β*-Gal^HI^ fraction that displayed an MFI of 15.1×10^3^. The remaining 50% of less intensely fluorescent cells were sorted into a second fraction, SA-*β*-Gal^LO^, with MFI of 5.9×10^3^. SA-*β*-Gal fluorescence was not proportional to apparent cell size (Figure 1b), suggesting that SA-*β*-Gal may be specific to senescence rather than a reporter of cellular hypertrophy.

To evaluate the SA-*β*-Gal^HI^ cells for additional features of senescence, the sorted cells were returned to culture for 2 days, then examined by microscopy and stained for additional markers of senescence. The SA-*β*-Gal^HI^ cells were nearly homogeneous, displaying the characteristic flattened shape and enlarged cell size of senescent cells with nuclei marked by senescence-associated heterochromatin foci (SAHF), increased immunoreactivity and delocalization of CDK inhibitor proteins p16^Ink4A^ and p21^Cip1^, and a disordered cytoskeleton with cytoplasmic filamentous (F) actin and nuclear monomeric (G) actin (Figure 1c). These data validate FACS as a means to obtain highly enriched populations of viable senescent cells, enabling biochemical and molecular analysis.

### Tandem mass spectrometry analysis yields a TIS proteome

Toward identifying proteins associated with therapy-induced senescent arrest, FACS was again used to enrich SA-*β*-Gal^HI^ (S) and SA-*β*-Gal^LO^ (L) cells from etoposide-treated B16-F10 cells (Figure 1d). In parallel, FACS was used to isolate fractions from B16-F10 cultures that were actively proliferating (P) or had reached confluence and become quiescent (Q). Each population of cells was lysed and separated into cytoplasmic, nuclear, and membrane-bound fractions. The proteins were separated by denaturing polyacrylamide gel electrophoresis, the gels divided into sections and subjected to in-gel trypsinization. Purified peptides were analyzed by Orbitrap LC-MS/MS and the spectra matched to peptides and proteins by MaxQuant database search. Then, protein abundance was compared across samples by MaxQuant label-free quantitation (LFQ).

**Figure 2:**
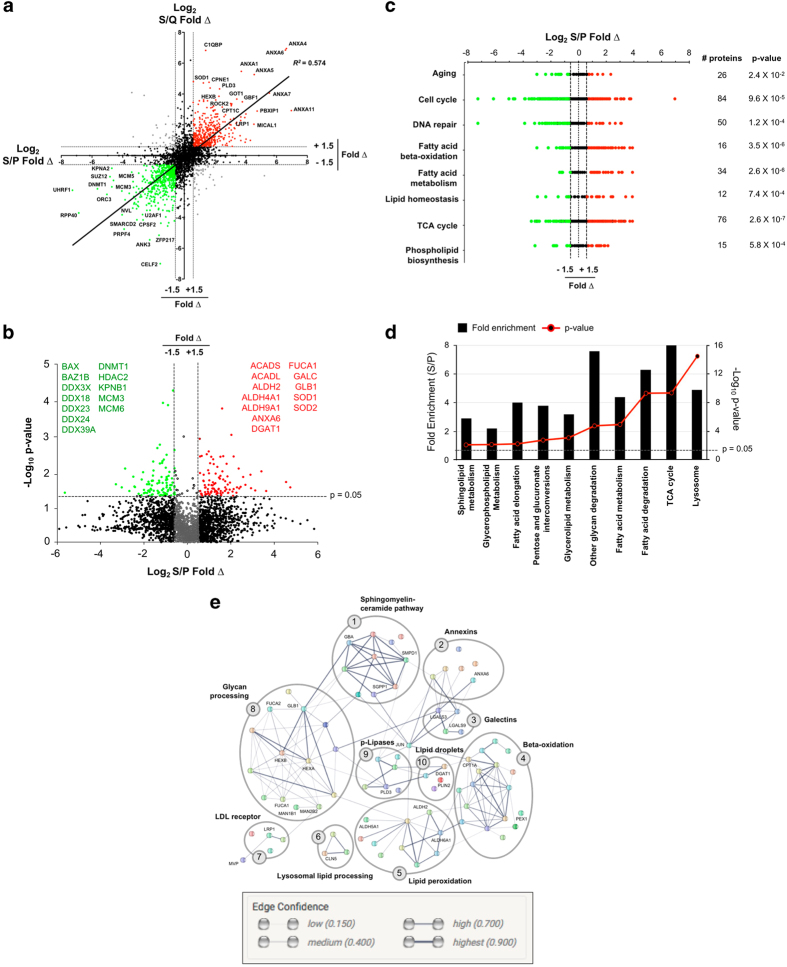
Proteomics analysis of FACS-enriched senescent cells suggests dysregulation of biological pathways involved in lipid hometostasis. (**a**) Quadrant plot showing significantly changed protein ratios for senescence *versus* proliferation (S *versus* P, *x*-axis) and senescence *versus* quiescence (S *versus* Q, *y*-axis). Log_2_ ratio values shown. Many proteins display similarly altered expression in senescence compared to proliferation or quiescence. Solid line: linear regression trendline, determined using significantly changed protein data points (red, green); *R*^*2*^=0.574. (**b**) Volcano plot visualizing fold change (log_2_) plotted *versus p*-value (−log_10_) for the ratio of protein expression in FACS-enriched senescent cells (S) *versus* proliferating cells (P). *P*-values were calculated using mean label-free quantitation spectral intensity data for statistical analysis using a paired, two-tailed *t*-test. Protein hits meeting both fold change and statistical significance are indicated in green (downregulated) or red (upregulated). Proteins of particular interest are named on the plot. (**c**) GO categories enriched for significantly changed levels of proteins for senescence *versus* proliferation (S *versus* P). Enriched GO categories included terms conventionally associated with senescence and several related to lipid homeostasis. Green, negative change; red, positive change during senescence. Number of proteins per GO term and *p*-value of term enrichment are shown to right of dot plot. (**d**) KEGG biological pathways related to lipid homeostasis and glycolipid processing found to be significantly upregulated in senescence, as evidenced by both fold change (black) and *p*-values (red). (**e**) Protein network analysis demonstrates interconnections of lipid homeostasis pathways found to be enriched in accelerated senescence. STRING database protein network analysis showing literature-reported interactions between lipid-related proteins identified as upregulated during senescence in this study. Interaction confidence levels are indicated by line weight, as shown. Ten subcategories were identified, from (1) sphingomyelin-ceramide pathway to (10) lipid droplets.

In total, 3590 mouse proteins were identified at 1% false discovery rate, of which 3190 appeared in at least 2 replicates. Of these, 2886 proteins were identified in SA-*β*-Gal^HI^, 2502 in SA-*β*-Gal^LO^, 2970 in proliferating and 2559 in quiescent cells. To highlight senescence-specific changes in protein abundance, Log_2_ ratios were compared between protein LFQ intensities in SA-*β*-Gal^HI^ (S) cells compared to proliferating (P) or quiescent (Q) cells in a quad plot (Figure 2a) and to SA-*β*-Gal^LO^ (L) cells (Supplementary Table 1). Overall, the protein abundance log_2_ ratios displayed a moderate correlation between S/P and S/Q (*R*^2^=0.574), with 428 proteins (14.5%) significantly upregulated (log_2_ S/P, S/Q⩾1.5) and 475 (16.2%) downregulated (log_2_ S/P, S/Q⩽1.5). LFQ intensities in SA-*β*-Gal^LO^ (L) were also compared to proliferating (P) cells (Supplementary Figure 2).

To identify changes in protein expression most specific to senescence, mean S/P LFQ intensity ratios were subjected to a paired, two-tailed *t*-test. Fold-change (log_2_) and *p*-value (−log_10_) were co-visualized for each protein using a volcano plot (Figure 2b). Although 62.6% of proteins displayed 1.5-fold log_2_ change in expression levels, only 5.8% of total proteins were found to exhibit statistically significant (*p*⩽0.05) change in expression, and only 5.3% met both criteria. Proteins downregulated in senescence included apoptosis regulator BAX, tyrosine kinase BAZ1B, DDX family DNA helicases, DNA methyltransferase DNMT1, histone deacetylase HDAC2, karyopherin KPNB1 and two MCM family DNA replication proteins, MCM3 and MCM6. Upregulated proteins included fatty acid beta-oxidation enzymes ACADS, ACADL, aldehyde dehydrogenases including ALDH2, ALDH4A1, and ALDH9A1, annexin phospholipid binding protein ANXA6, triglyceride synthesis enzyme DGAT1, glycolipid processing enzymes FUCA1, GALC, GLB1, GUSB, and NAGLU, and redox regulators including SOD1 and SOD2.

### Upregulation of glycolipid processing during TIS

Further analysis of differentially expressed proteins with the DAVID Bioinformatics Database identified enriched GO terms (Figure 2c) while mining the KEGG revealed the most altered biological pathways (Figure 2d). Along with expected GO terms cell cycle ([GO:0007049], *p*=9.6×10^−5^), DNA repair ([GO:0006281], *p*=1.2×10^−4^), and aging ([GO:0007568], *p*=2.4×10^−2^), 24 GO terms related to cellular lipid regulation were also identified including fatty acid beta-oxidation ([GO:0006635], *p*=3.5×10^−6^), fatty acid metabolism ([GO:0006631], *p*=2.6×10^−6^), and lipid homeostasis ([GO:0055088], *p*=7.4×10^−4^). In total, 24 significantly enriched GO terms related to cellular lipid regulation were found (Supplementary Table 2). Similarly, KEGG analysis identified 10 biological pathways related to cellular lipid processing and metabolism that displayed ⩾2.0-fold enrichment and *p*-value ⩽0.05 (Figure 2d), including sphingolipid metabolism (*p*=2.0×10^−2^), fatty acid metabolism (*p*=2.5×10^−5^), and lysosome (*p*=4.5×10^−15^). Protein interaction analysis using the STRING protein network database revealed many known protein associations with STRING scores indicating medium to highest confidence linking lipid-related proteins identified by GO and KEGG (Figure 2e). Clusters of proteins include galectins, glycan processing, sphingolipid-ceramide metabolism, annexins, fatty acid beta-oxidation, lipid droplets (LD), LDL receptor, lysosomal lipid processing, phospholipases, and lipid peroxidation.

**Figure 3:**
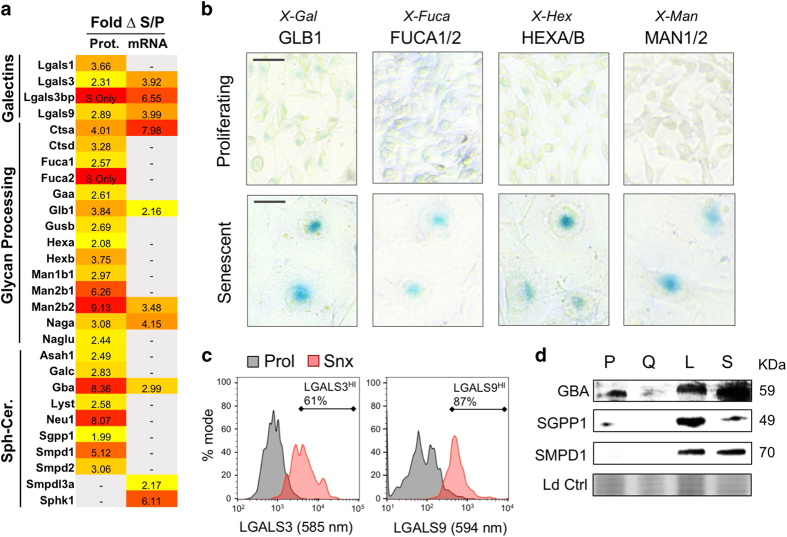
Upregulation of glycolipid processing proteins during accelerated senescence. (**a**) Heat map showing proteomics and transcriptomics data for glycolipid processing genes upregulated in senescence, including subcategories of galectins, glycan processing proteins, and sphingolipid-ceramide metabolism. (**b**) Chromogenic staining of glycolipid processing enzymes GLB1, FUCA1/2, HEXA/B, and MAN1/2 using indolyl enzyme substrates as indicated. (**c**) Flow cytometric analysis of cell surface expression of galectins LGALS3 and LGALS9 on etoposide-treated senescent cells (S, red) and proliferating cells (P, black). (**d**) Western blot showing upregulation of sphingolipid-ceramide pathway proteins GBA, SGPP1, and SMPD1 in senescent (S) compared to proliferating (P) and quiescent (Q) cells. Equal loading indicated by total protein stain (Ld Ctrl).

A complementary transcriptomics analysis of FACS-enriched senescent and proliferating cells revealed 1266 differentially expressed genes, of which 158 were also identified by proteomics (Supplementary Figure 3). Multiple glycolipid processing proteins were overexpressed in senescence, including galectins, glycan processing proteins, and sphingolipid-ceramide metabolic pathway enzymes. Several displayed increased abundance at both the protein and messenger RNA (mRNA) levels, including LGALS3 (2.3 fold-change protein, 3.9 fold-change RNA), LGALS3BP (senescent cells only; 6.6), LGALS9 (2.9, 4.0), CTSA (4.0, 8.0), GLB1 (3.8, 2.2), MAN2B2 (9.1, 3.5), NAGA (3.1, 4.2), and GBA (8.4, 3.0, Figure 3a).

Notably, the standard SA-*β*-Gal assay for detection of senescence uses the chromogenic enzyme substrate *X-Gal* to detect activity of the glycolipid processing beta-galactosidase GLB1 (Figure 3b, left). Analogous indolyl substrates are similarly available to detect several of the other upregulated glycosidases, including *X-Fuca* for FUCA1/2, *X-Hex* for HEXA/B, and *X-Man* for MAN1/2. Like *X-Gal*, *X-Fuca*, *X-Man*, and *X-Hex* yielded characteristic blue staining in >90% of senescent cells, confirming upregulation of multiple glycolipid processing enzymes (Figure 3b).

Galectins are galactose-binding proteins often expressed on the extracellular surface of cell membranes, making them potentially useful surface markers for following senescence in living cells. Flow cytometry revealed increased expression of LGALS3 and LGALS9 on senescent cells compared to proliferative cells (Figure 3c).

Several sphingolipid-ceramide pathway proteins were identified by proteomics analysis, including GALC, GBA, NEU1, SGPP1, SMPD1, and SPHK1. Western blotting for GBA, SMPD1, and SGPP1 confirmed upregulation in SA-*β*-Gal^HI^ (S) and/or SA-*β*-Gal^LO^ (L) cells compared to proliferating and quiescent cells (Figure 3d). The lysosomal acid sphingomyelinase SMPD1 appeared to be the most specific to senescence.

### Altered lipid homeostasis in TIS

**Figure 4:**
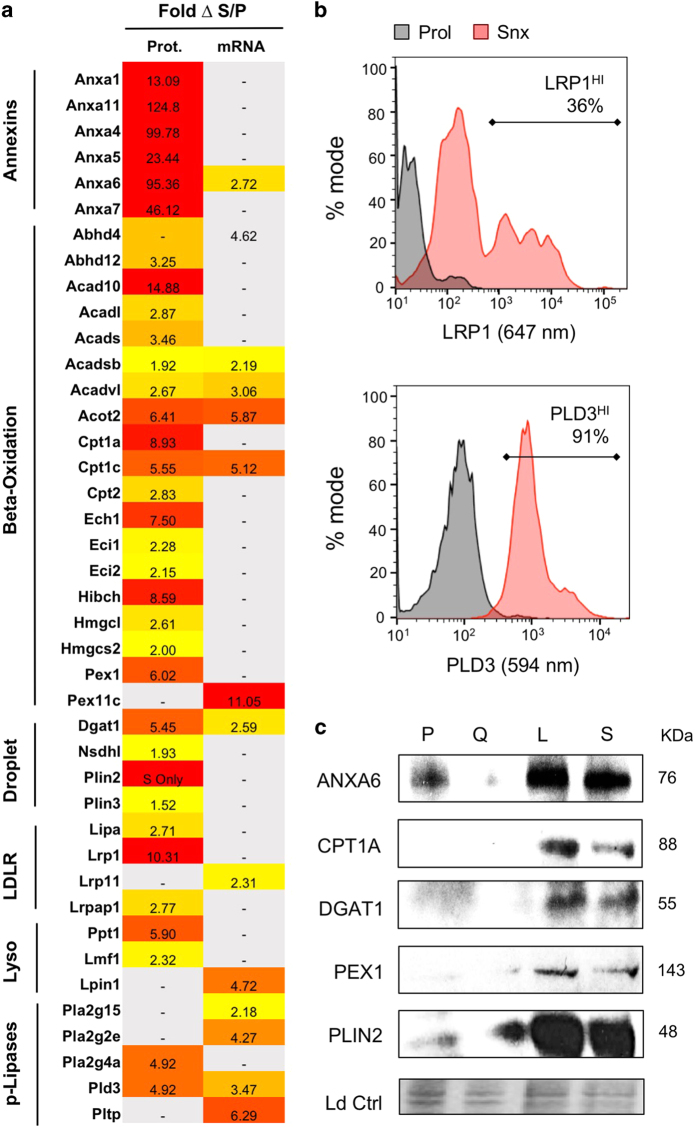
Overexpression of proteins involved in cellular lipid homeostasis in accelerated senescence. (**a**) Heat map showing proteomics and transcriptomics data for lipid homeostasis related genes upregulated in senescence, including subcategories of annexins, fatty acid beta-oxidation, LDs, LDL receptor, lysosomal lipid processing, and phospholipases. (**b**) Flow cytometric analysis of lipid metabolism-related cell surface proteins LRP1 and PLD3 in proliferating (P, black) and senescent (S, red) cells. (**c**) Western blotting data confirming upregulation of lipid associated proteins ANXA6, CLN5, CPT1A, DGAT1, PEX1, and PLIN2 in senescent (SA-*β*-Gal^LO^, L; SA-*β*-Gal^HI^, S) compared to proliferating (P) and quiescent (Q) cells. Equal protein loading indicated by total protein stain.

Proteomic analysis revealed upregulation of over 40 lipid regulatory proteins in senescence, including annexins and proteins related to fatty acid beta-oxidation, LD, LDL receptor, lysosomal lipid processing, and several phospholipases (Figure 4a). Upregulation of both protein and mRNA was observed for ANXA6 (95.4-fold-change protein, 2.7 fold-change RNA), ACADSB (1.9, 2.2), ACADVL (2.7, 3.1), ACOT2 (6.4, 5.9), CPT1C (5.6, 5.1), DGAT1 (2.6, 5.5), and PLD3 (4.9, 3.5). Flow cytometry detected increased surface expression of low-density lipoprotein receptor component protein LRP1 (CD91) and phospholipase D3 (PLD3) (Figure 4b) while western blotting analysis revealed increased expression of lipid regulatory proteins ANXA6, CPT1A, DGAT1, PEX1, and PLIN2 (Figure 4c).

**Figure 5:**
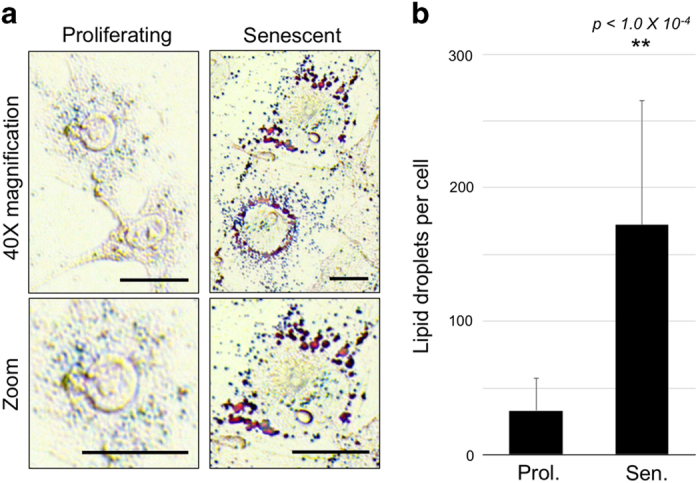
Lipid storage droplets are abundant and enlarged in senescent cells. (**a**) Oil Red O staining reveals larger and more numerous LDs in senescent cells. Scale bar=10 *μ*m. (**b**) Enumeration of LDs per cell (mean and S.D. indicated, **p<1.0×10^−4^ by unpaired, two-tailed *t*-test, *n*=20 cells).

**Figure 6:**
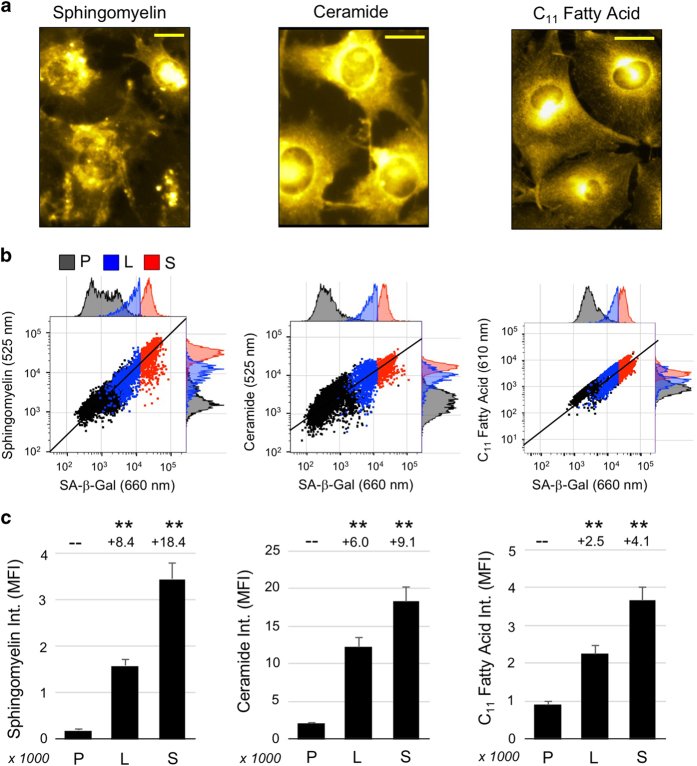
Upregulation of lipid import is correlated with SA-*β*-Gal overexpression in TIS. (**a**) Images of etoposide-induced senescent cells incubated with fluorescent sphingomyelin, ceramide, or C_11_ fatty acid for 30 min. Scale bar=10 *μ*m. (**b**) Flow cytometry analysis of uptake for each fluorescent lipid *versus* SA-*β*-Gal, examined in proliferating (P, black) or etoposide-induced senescent cells, indicating SA-*β*-Gal^LO^ (L, blue) and SA-*β*-Gal^HI^ (S, red) populations. General correlation of SA-*β*-Gal expression *versus* lipid uptake can be seen, as indicated by solid black line. (**c**) MFI data for cell populations shown in **b**. Fold-increase in MFI of SA-*β*-Gal^LO^ (L) or SA-*β*-Gal^HI^ (S) *versus* proliferating cells (P) is shown at top of graph, **p<1.0×10^−4^ by unpaired, two-tailed *t*-test, *n*=500 cells.

The pattern of altered gene expression raised the question whether senescent cell phenotypes might include deregulation of lipid metabolism. Oil Red O staining revealed abundant LDs in senescent cells (Figure 5a) with a mean of 172 LDs per cell compared to 33 LDs per cell for proliferating cells (*n*=20, *p*<1.0×10^−4^ by unpaired *t*-test, Figure 5b). To assess the potential of extracellular lipid import by senescent cells, fluorescent analogs of sphingomyelin, ceramide, and C_11_ fatty acid were applied to cells for 30 min and cells examined by microscopy and flow cytometry (Figure 6). Phosphocholine and cholesterol yielded similar results (Supplementary Figure 4). In each case, lipid uptake appeared to correlate with SA-*β*-Gal activity. Compared to proliferating cells, SA-*β*-Gal^HI^ cells took up 18.4-fold more sphingomyelin, 9.1-fold more ceramide, and 4.1-fold more C_11_ fatty acid (*p*<1.0×10^−4^ for each).

**Figure 7:**
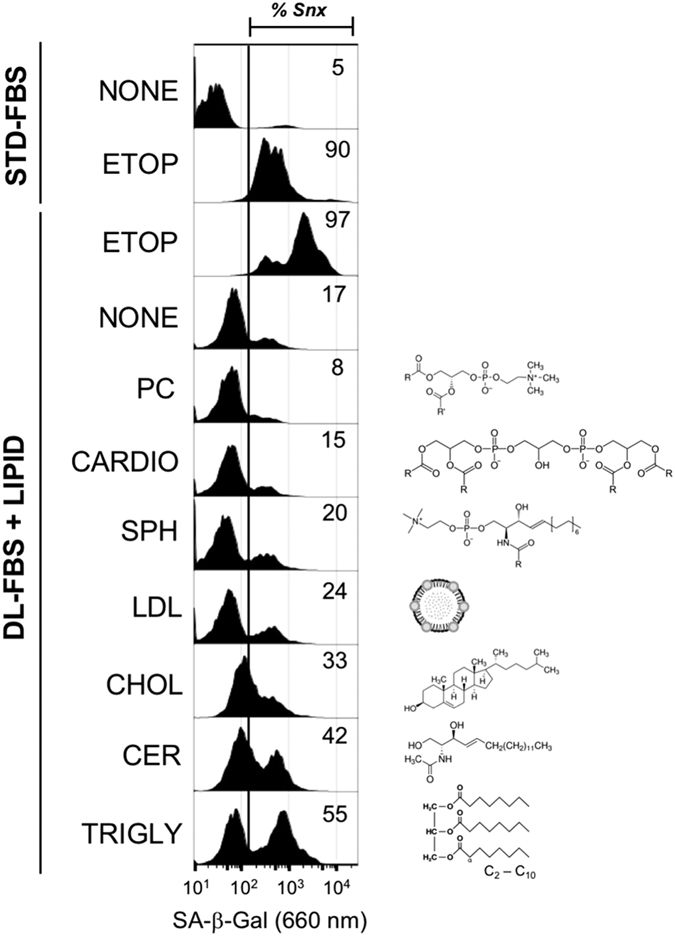
Ceramide and triglyceride induce accelerated senescence. B16-F10 tumor cells were cultured in media supplemented with standard or delipidized FBS for 72 h. Etoposide (ETOP) induced a similar, high percentage of SA-*β*-Gal^+^ cells in both media. Addition of phosphatidylcholine (PC), cardiolipin (CARDIO), sphingomyelin (SPH), low-density lipoprotein (LDL), or cholesterol (CHOL) to the DL-FBS had a small effect on SA-*β*-Gal. Adding C_2_-ceramide (CER) or a C_2_-C_10_ triglyceride mixture (TRIGLY) enhanced expression of SA-*β*-Gal, indicating that these lipids effectively induced accelerated senescence.

These results raised the question whether modulating lipid uptake might be sufficient to influence onset of cellular senescence. Thus, we cultured B16-F10 murine melanoma cells in delipidized fetal bovine serum (FBS) media, spiking in phosphatidylcholine, cardiolipin, sphingomyelin, low-density lipoprotein, cholesterol, C_2_-ceramide, or a triglyceride mixture, and then assayed senescence by flow cytometry using the DDAOG SA-*β*-Gal assay (Figure 7). Culture in delipidized media alone for 72 h was sufficient to shift 17% of the cells to senescence. Treating cells cultured in delipidized FBS media with etoposide efficiently induced senescence (97% of cells), even more than for cells in media with standard FBS (90%). Surprisingly, addition of specific lipids to the delipidized media was sufficient to significantly increase cellular senescence, including cholesterol (33%), ceramide (42%), and triglycerides (55%). These results suggest that altered lipid metabolism on its own might activate the senescence program.

### Lipid peroxidation in TIS

**Figure 8:**
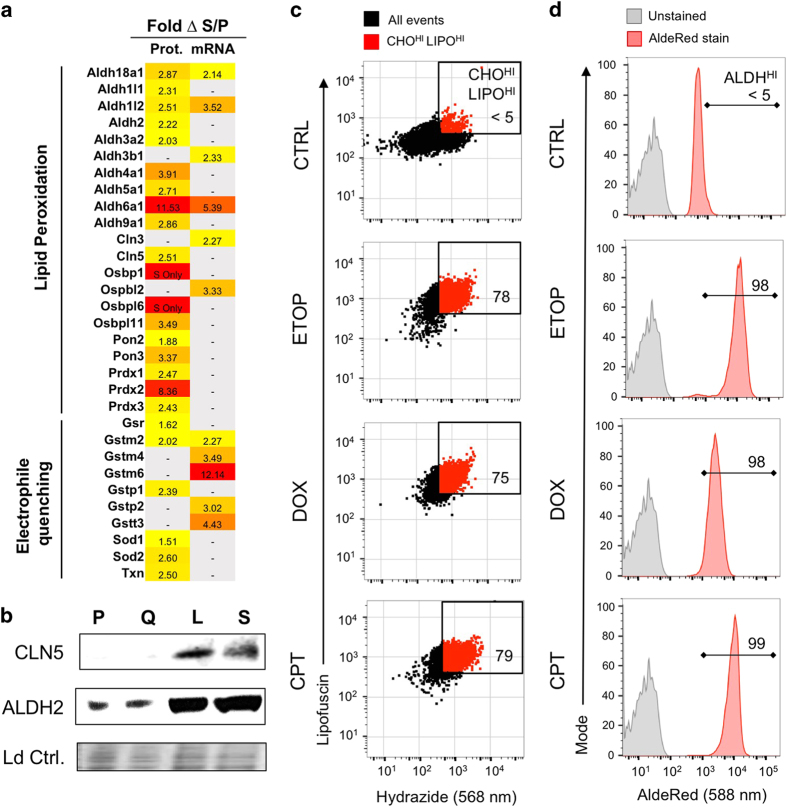
Therapy-induced senescence is characterized by increased cellular aldehydes and elevated aldehyde dehydrogenase activity. (**a**) Heat map showing proteomics and transcriptomics data for lipid peroxidation related genes found to be overexpressed in senescence, including subcategories of lipid peroxidation and electrophile quenching. (**b**) Western blot showing expression of lipofuscinosis related protein CLN5 and mitochondrial aldehyde dehydrogenase ALDH2 in proliferating (P), quiescent (Q), SA-*β*-Gal^LO^ (L) or SA-*β*-Gal^HI^ (S) senescent cells. Equal protein loading indicated by total protein stain (Ld Ctrl). (**c**) Flow cytometry assay using Alexa 568 hydrazide for aldehyde levels in cells treated with topoisomerase poisons etoposide (ETOP), doxorubicin (DOX), or camptothecin (CPT). For each agent, aldehyde detection by Alexa 568 is plotted *versus* lipofuscin autofluorescence, a senescence marker associated with lipid peroxidation. A gate drawn to identify high aldehyde, high lipofuscin cells, based on the signal from the proliferating cell control (<5%) indicates high aldehyde levels correspond with lipofuscin accumulation in TIS. (**d**) Flow cytometry assay with AldeRed-588 for ALDH enzyme activity in cells treated with topoisomerase poisons etoposide (ETOP), doxorubicin (DOX), or camptothecin (CPT). An unstained reference sample is shown in gray. A gate drawn based on the proliferating cell control indicates increased ALDH activity in TIS, with the percentage of ALDH^HI^ cells indicated on each histogram.

A potential mechanistic link between altered lipid metabolism and cellular senescence is provided by recent work from our group^15^ implicating aldehyde end-products of lipid peroxidation such as 4-hydroxynonenal as key mediators of TIS. Indeed, the proteomics analysis revealed multiple upregulated proteins related to lipid peroxidation, aldehyde detoxification, and response to oxidative stress (Figure 8a). Proteins that enhance cellular adaptation to lipid peroxidation included ten aldehyde dehydrogenases (ALDH protein family), two lysosomal palmitoyl-protein thioesterases (CLN proteins), four oxysterol binding proteins (OSBPs), and two serum paraoxonases (PONs). Upregulated proteins involved in detoxifying free radicals, electrophiles and other reactive species included gluthathione pathway proteins (GSR, GST), superoxide dismutases (SOD1 and 2) and thioredoxin. Several of these proteins were upregulated at both the proteomic and mRNA level including ALDH18A1 (2.9 fold-change protein, 2.1-fold-change RNA), ALDH1L2 (2.5, 3.5), ALDH6A1 (11.5, 5.4), and GSTM2 (2.0, 2.3). Western blotting for CLN5 and ALDH2 confirmed upregulation in senescence (Figure 8b).

To extend our prior studies linking senescence to lipid peroxidation and production of lipid aldehydes, we examined B16-F10 cells treated with etoposide, doxorubicin, or camptothecin by flow cytometry to simultaneously evaluate the fluorescent lipid peroxidation marker lipofuscin and the aldehyde-reactive probe Alexa Fluor 568 Hydrazide. Compared to untreated, non-senescent control cells, senescent cells induced by all three agents displayed both high lipofuscin and high cellular aldehydes (Figure 8c). In turn, flow cytometric analysis of senescent cells induced by etoposide, doxorubicin, or camptothecin revealed a marked increase in ALDH activity based on activation of the fluorescent probe AldeRed-588 (Figure 8d).

## Discussion

Despite many years of study, cell senescence remains a somewhat enigmatic cell state. Whether induced by replicative, oncogenic, or therapeutic stress, senescence develops slowly in a subset of cells, and in competition with cell cycle arrest, cell death, and proliferation, resulting in heterogeneous populations of cells. Under optimal conditions, most surviving cells will display a characteristic pattern of cellular features consistent with senescence. Senescence is often evaluated by the SA-*β*-Gal assay which reports on the activity of the beta-galactosidase GLB1. Overcoming limitations of prior assays, we recently described DDAOG (9H-(1,3-dichloro-9,9-dimethylacridin-2-one-7-yl) *β*-D-galactopyranoside) as a live-cell fluorescent SA-*β*-Gal reporter.^15^ Here, we explored using DDAOG to isolate senescent cells for molecular analysis.

Thus, tumor cells treated for five days with the topoisomerase II poison etoposide and stained with DDAOG were sorted to isolate enriched populations of viable senescent cells. Along with high SA-*β*-Gal activity, the cells displayed the flattened morphology, heterochromatin foci, overexpression of the p16 and p21 cell cycle inhibitory proteins, and altered actin cytoskeleton characteristic of senescence. Label-free quantitative proteomic analysis of the SA-*β*-Gal^HI^ fraction revealed a distinct pattern of protein expression that comprised a candidate TIS proteome. Systems biology analysis of GO terms revealed that along with the expected enrichment of senescence-associated pathways including aging, cell cycle, and DNA damage, the data mapped to multiple terms related to glycolipid processing, lipid homeostasis, and lipid peroxidation.

Despite its use as a specific senescence marker, previous work has not revealed how GLB1 activation may be linked to senescent cell formation or survival. GLB1 is one of several enzymes that localize in the endo-lysosomal compartment and cleave glycans. Much like GLB1, we observed upregulation of other glycan cleaving enzymes including the mannosidases MAN1B1 and MAN2B1, hexosaminidases HEXA and HEXB, and fucosidases FUCA1, FUCA2 at the proteomic and/or transcriptomic level. In turn, chromogenic staining using indolyl-based cleavable enzyme substrates X*-Fuca*, *X-Hex*, and *X-Man* confirmed these results, potentially expanding the toolbox of fixed-cell senescence assays beyond *X-Gal*. Toward additional markers that can be used to identify living senescent cells, proteomic analysis identified several membrane-associated proteins with links to lipid metabolism whose overexpression at the cell surface could be detected by flow cytometry, including LRP1/CD91, phospholipase D3 (PLD3), and galectins LGALS3 and LGALS9.

Along with GLB1, other glycosphingolipid degradation enzymes including GBA, SMPD1, and SGPP1 were upregulated in TIS. GLB1 cleaves lactosylceramides to produce glucosylceramides, which in turn are processed by glucosylceramidase (GBA) to form ceramide.^21^ Ceramide levels are also regulated by sphingomyelin phosphodiesterase (SMPD1) and sphingosine phosphate phosphodiesterase 1 (SGPP1).^22^ Mutations in GLB1 result in a lysosomal storage disease associated with accumulation of the glycosphingolipid GM1 ganglioside.^23^ GBA mutation causes lysosomal glucosylceramide accumulation, leading to Gaucher disease with association to Parkinson’s disease,^24^ while SMPD1 deficiency causes sphingomyelin accumulation and Niemann-Pick disease.^25^ One hypothesis is that upregulation of glycosphingolipid degradation may be an adaptive response in TIS. Indeed, we observed enhanced uptake of fluorescent ceramide and sphingolipid tracers in senescent cells, consistent with higher flux through the sphingolipid pathway and increased cellular ceramide. Strikingly, culturing tumor cells with extracellular ceramide as their sole lipid source was sufficient to induce senescence, confirming a special role for ceramide metabolism in accelerated senescence.

Systems biology analysis highlighted a pattern of upregulation of lipid processing and metabolism in TIS cells. Proteins and pathways involved in lipid binding, import, signaling, biosynthesis, degradation, and storage were prominent. Overexpression of LD marker perilipin-2 (PLIN2), along with increased LDs detected with Oil Red O staining, supports an overall accumulation of lipids in TIS cells. While the overexpression of DGAT1 may suggest a net increase in triglyceride synthesis, lipid import likely also contributes to accumulation, given the overexpression of the LDL receptor protein LRP1/CD91, the enhanced uptake of fluorescent lipids as well as the senescence induction by triglycerides. Downstream, we observed upregulation of peroxisomal (PEX1/11c) and mitochondrial (for example, ACADL/S/VL, ACOT2, CPT1A/C, CPT2) fatty acid beta-oxidation regulators at both the proteomic and transcriptional level. Upregulation of PEX1 indicates increased peroxisomal biogenesis, which facilitates long chain fatty acid breakdown. CPT1A mediates the carnitine shuttle, which transports the resulting medium chain fatty acids into mitochondria for the final, energy-producing steps of beta-oxidation.

A common feature of beta-oxidation whether in peroxisomes or mitochondria is the potential to generate reactive oxygen species. Thus, lipid metabolism itself may be responsible for the increased ROS we detected in senescent cells. Consistent with oxidative stress, we observed upregulation of glutathione biosynthesis (GSR, GST), superoxide dismutases (SOD1/2), and thioredoxin (TXN) in senescent cells. Oxidative stress is a well-known inducer of senescence, typically acribed to ROS-mediated damage to metabolites, proteins and/or nucleic acids. However, our recent work^15^ suggests that lipids may be the critical target. ROS can induce lipid peroxidation, which initiates a free-radical cascade that terminates via formation of lipid aldehyde end-products (as reviewed in Ayala *et al.*^18^ and Gueraud^26^). Using a cell-permeant, aldehyde-reactive probe, we detected increased cellular aldehydes accumulating after treatment with etoposide, doxorubicin, or camptothecin. Accumulation of lipid aldehydes results in DNA and protein adducts,^27–29^ altering gene expression, protein activity, and cell signaling, and further contributing to a persistent condition of cell stress and damage. Upregulation of neuronal ceroid lipofuscinosis disease related proteins CLN3/5 may reflect oxidized lipoprotein aggregates,^30^ while increased oxysterol binding proteins OSBP1/2/6/11 may indicate oxidized cholesterol,^31^ and increased serum paraoxonases PON2/3 may antagonize lipid peroxidation at the plasma membrane.^32^ In the short term, glutathione can sequester lipid aldehydes but is depleted by chronic aldehyde exposure.^33^ Lipid aldehydes activate the Keap1/Nrf2 antioxidant response,^34^ increasing expression of aldehyde dehydrogenases.^35,36^ Indeed, induction of ALDH gene expression was detected at both the transcriptional and protein levels. Increased ALDH enzyme activity was also detected, indicating that aldehydes had likely activated the ALDH.^36^

In this study, we used the chemotherapy agents etoposide, doxorubicin, and camptothecin to promote TIS. These agents are generally considered to promote genotoxic stress via poisoning topoisomerases.^37^ However, this study and our recent work examining lipid aldehydes as inducers of senescence^15^ point to impacts of topoisomerase poisons on lipid metabolism that are likely independent of their established mechanisms of action that involve binding to topoisomerases. That each of these compounds increases cellular ROS^38–40^ provides a potential source of free radicals to initiate lipid peroxidation and drive formation of lipid aldehydes. Suggesting that this alternative activity may be of primary importance to TIS, our prior work^15^ showed that sequestering lipid aldehydes was sufficient to prevent etoposide, doxorubicin, camptothecin, and other topoisomerase poisons from inducing SA-*β*-Gal or other markers of senescence. Taken together, we infer that lipid peroxidation and/or lipid aldehydes may also drive the increased lipid import, accumulation, and metabolism we observed in cells expressing high SA-*β*-Gal activity. Thereby, deregulation of lipid metabolism may initiate a positive feedback loop that serves to maintain senescent arrest, independent of the persistence of DNA damage or other initiating stresses.

## Materials and methods

### Cell culture

Cell lines B16-F10 (murine metastatic melanoma), A375-MA2 (human metastatic melanoma), and A549 (human lung adenocarcinoma) were obtained from a commercial cell bank (American Type Culture Collection, Manassas, VA, USA) and grown in culture medium (DMEM-HI, #11960, Life Technologies, Grand Island, NY, USA) supplemented with 10% FBS (Gemini Biosciences, West Sacramento, CA, USA), 4 mM l-glutamine (Gemini Biosciences), and 1X penicillin–streptomycin solution (Life Technologies). Cells were manipulated under sterile conditions and grown in a humidified incubator at 37 °C with 5% CO_2_ for <10 passages (~30 population doublings). Prior to senescence assays, cells were plated at low density (5×10^3^ per cm^2^) in culture vessels and incubated overnight (18 h). Etoposide (2.0 *μ*M; Sigma-Aldrich, St Louis, MO, USA) was then added to cultures for 96 h to induce TIS. Alternatively, doxorubicin (0.05 or 0.10 *μ*M; Sigma-Aldrich) or camptothecin (0.05 or 0.10 *μ*M; Sigma-Aldrich) were used. Untreated samples were grown to confluence as quiescent cell controls. Actively dividing cells were plated at low density (5×10^3^ per cm^2^) and cultured 24 h as proliferating cell controls.

### Flow cytometric sorting of senescent cells

Senescent, quiescent, and proliferating cell cultures were harvested by gentle mechanical dissociation of cell monolayers using sterile cell scrapers. Cells were transferred to conical tubes, pelleted by centrifugation at 1200×*g* for 5 min, resuspended in 1 ml of 1% BSA-DPBS, and counted using a brightfield hemacytometer. 10×10^6^ cells per condition were transferred to conical tubes, pelleted by centrifugation, and resuspended in 10 ml of DMEM-HI culture medium without FBS or other supplements. Bafilomycin A1 (Research Products International, Mt. Prospect, IL, USA) was added at 1 *μ*M for 30 min to alkalinize lysosomal pH for subsequent detection of SA-*β*-Gal using DDAOG (10 *μ*g/ml, Life Technologies). Cells were stained with DDAOG for 1 h at 37 °C in an incubator without CO_2_, washed once in 10 ml of 1% BSA-DPBS, and then resuspended in sterile, ice-cold FACS buffer consisting of 1X DPBS, 10 mM HEPES, 4.5 mg/ml glucose, 4 mM l-glutamine, 1% BSA, and 2 mM EDTA.

Senescent cells were detected and sorted with a BD Aria III using FACSDiva software, based on DDAO fluorescence using 640 nm excitation and 670 nm emission into SA-*β*-Gal^LO^ and SA-*β*-Gal^HI^ fractions. The instrument was calibrated using fluorescent calibration and drop delay particles (SpheroTech, Lake Forest, IL, USA) and SA-*β*-Gal background was determined using proliferating and quiescent samples, which were also sorted using scatter gating as controls. Dead cells and debris were eliminated using FSC *versus* SSC gating (Supplementary Figure 7). Data in.fcs listmode were analyzed with FlowJo software (FlowJo LLC, Ashland, OR, USA) to plot results and perform statistical analysis.

### Mass spectrometry analysis

Cells sorted into complete culture medium were allowed to recover for 1 h, then pelleted by centrifugation. The supernatant was removed and cell pellets were snap-frozen in liquid nitrogen. For each cell sample, cells were thawed and whole-cell lysate prepared from at least 1×10^6^ cells. A Subcellular Protein Fractionation Kit (Life Technologies) was used according to manufacturer's instructions. For samples S (SA-*β*-Gal^HI^) and P (proliferating), two biological and two technical replicates were performed. For samples L (SA-*β*-Gal^LO^) and Q (quiescent), one biological replicate and two technical replicates were performed.

20 *μ*g of the cytoplasmic, nuclear, or membrane fraction of each lysate was loaded onto a 12% Bis-Tris acrylamide SDS-PAGE gel with MOPS buffer (Life Technologies) and run for 10 min at 200 V constant. The gel was stained with Imperial Protein Stain (Life Technologies) at room temperature to visualize protein lanes. For each lane, 1 cm gel sections were excised by razor blade and cubed into ~1 mm^3^ gel sections. Each gel section was washed in dH_2_O and destained using 100 mM NH_4_HCO_3_ (pH 7.5; Sigma-Aldrich) in 50% acetonitrile (Sigma-Aldrich). A reduction step was performed by addition of 100 *μ*l 50 mM NH_4_HCO_3,_ (pH 7.5) and 10 *μ*l of 200 mM tris(2-carboxyethyl)phosphine HCl (TCEP; Pierce, Rockford, IL, USA) at 37 °C for 30 min. The proteins were alkylated by addition of 100 *μ*l of 50 mM iodoacetamide (Pierce) prepared fresh in 50 mM NH_4_HCO_3_ (pH 7.5), and allowed to react in the dark at 20 °C for 30 min. Gel sections were washed in water, then acetonitrile, and vacuum dried. Trypsin protein digestion was carried out overnight at 37 °C with 1 : 50 to 1 : 100 enzyme-protein ratio of sequencing grade modified trypsin (Promega, Madison, WI, USA) in 50 mM NH_4_HCO_3_ (pH 7.5), and 20 mM CaCl_2_ (Sigma-Aldrich). Peptides were extracted sequentially with 5% formic acid (Sigma-Aldrich), then with 75% acetonitrile in 5% formic acid, combined, and vacuum dried using a centrifugal concentrator (Eppendorf, Happaugue, NY, USA).

All samples were resuspended in HPLC-grade water (Honeywell—Burdick & Jackson, Muskegon, MI, USA) containing 0.2% formic acid, 0.1% trifluoroacetic acid (Sigma-Aldrich), and 0.002% Zwittergent 3–16 (EMD Millpore, Billerica, MA, USA), a sulfobetaine detergent that contributes the following distinct peaks at the end of chromatograms: MH^+^ at 392, in-source dimer [2 M+H^+^] at 783, and some minor impurities of Zwittergent 3–12 seen as MH^+^ at 336. The peptide samples were loaded to a 0.25 *μ*l C_8_ OptiPak trapping cartridge custom-packed with Michrom Magic C_8_ (Optimize Technologies, Oregon City, OR, USA), washed, then switched in-line with a 20 cm×75 *μ*m C_18_-packed spray tip nanocolumn packed with Michrom Magic C_18_ AQ (Optimize Technologies), for a two-step gradient. Mobile phase A was H_2_O/acetonitrile/formic acid (98/2/0.2) and mobile phase B was acetonitrile/isopropanol/water/formic acid (80/10/10/0.2). Using a flow rate of 0.350 *μ*l/min, a 90 min, two-step LC gradient was run from 5 to 50% B in 60 min, followed by 50–95% B over the next 10 min, held 10 min at 95% B, returned to starting conditions, and re-equilibrated.

The samples were analyzed via electrospray tandem mass spectrometry (LC-MS/MS) on a Thermo Q-Exactive Orbitrap mass spectrometer, using a 70 000 RP survey scan in profile mode, *m*/*z* 360 to 2000 Da, with lockmasses, followed by 20 MS/MS HCD fragmentation scans at 17 500 resolution on doubly and triply charged precursors. Singly charged ions were excluded, and ions selected for MS/MS were placed on an exclusion list for 60 s. All LC-MS/MS *.raw data files were analyzed with MaxQuant (Max Planck Institute, V. 1.5.2.8) searching against the SPROT *Mus musculus* database (160223_SPROT_Mus_Iso_AUP000000589.fasta, UniProt, 2016). The following analysis criteria were used: LFQ was selected with a minimum of 1 high confidence peptide to assign quantitation intensities. Trypsin was identified as the protease with maximum missed cleavage set to 2. Carbamidomethyl (C) was selected as a fixed modification. Variable modifications were set to oxidization (M), formylation (N-terminal), deamidation (NQ), 4-hydroxynonenal HNE (CHK), and GlyGly (K). The Thermo Q-Exactive Orbitrap mass spectrometer was defined using an MS error of 20 ppm and a MS/MS error of 0.5 Da. 1% false discovery rate cutoff was selected for peptide, protein, and site identifications. Ratios were reported based on the MS level LFQ peak areas determined by MaxQuant and reported in the proteinGroups.txt file as LFQ intensities. Proteins were removed from this results file if they were flagged by MaxQuant as ‘Contaminants’, ‘Reverse’ or ‘Only Identified by Site’. A data filter was set such that identified protein hits were seen in at least two replicates.

This analysis produced a list of sorted proteomes of for the following sample states (P, proliferating; Q, quiescent, L, senescent, SA-*β*-Gal^LO^; S, senescent, SA-*β*-Gal^HI^). Ratios for (S/P), (L/P), (Q/P), (S/Q), and (S/L) were generated from average LFQ intensities of hits passing initial filtering criteria. Hits were considered significant if they exhibited ⩾1.5-fold change. *P*-values were determined from paired, two-tailed *t*-test results calculated from mean spectral count data. Mass spectrometry proteomic data have been deposited to the Proteomics Identifications (PRIDE) archive^41^ with the dataset DOI identifier 10.6019/PXD006216. A list of all proteins identified in our study and corresponding ratios can be found in Supplementary File 1 or by using PRIDE project accession number PXD006216.

### Transcriptomics analysis

To prepare samples for transcriptomics analysis, proliferating or FACS-enriched SA-*β*-Gal^HI^ cells were prepared as described above for two biological replicates per conditions. RNA was extracted using Trizol (Life Technologies) and purified using RNeasy mini kits (Qiagen, Valencia, CA, USA). Total RNA (1 *μ*g) was used for generation of cDNA and hybridized to an Illumina MouseRef 8 beadchip in duplicate (two technical replicates per condition). All cDNA probes, oligonucleotide microarray manipulations, and scanning of arrays were carried out by the Functional Genomics Core Facility at the University of Chicago. Data were analyzed by Illumina GenomeStudio using background correction and quantile normalization and filtered by detection of *p*-values ⩽0.05. Differentially expressed genes were detected with significance analysis of microarrays using a 2.0-fold cutoff and false discovery rate=0.01. The gene expression was then calculated by averaging probes using a custom Python script (Supplementary File 2).

### Fluorescent and brightfield microscopy analysis

Immunofluorescence imaging of SAHF, p16, p21, and F-/G-actin was performed on cells grown on sterile cover slips. Cell samples were washed in sterile 1x DPBS and fixed with fresh 4% paraformaldehyde (Electron Microscopy Sciences, Hatfield, PA, USA) for 10 min at 24 °C. Fixed cells were washed twice with DPBS and then permeabilized with 0.1% Triton-X-100 (Sigma-Aldrich) in DPBS for 5 min at 24 °C. For SAHF imaging, DAPI (Sigma-Aldrich) was applied at 1 *μ*g/ml in DPBS for 20 min at 24 °C, followed by a brief rinse with DPBS. For p16 and p21 imaging, rabbit monoclonal antibodies (Abcam, Cambridge, MA, USA) against each target were applied in 1% BSA-DPBS overnight at 4 °C, washed three times in 0.05% Triton-DPBS, incubated with anti-rabbit fluorescent secondary antibodies (DyLight 594, Life Techologies) in 5% BSA-DPBS for 30 min at 4 °C, and then washed again. For F- and G-actin detection, F-actin probe phalloidin Alexa Fluor 647 (1 unit, Invitrogen) and G-actin probe DNAse I Alexa Fluor 488 (0.3 *μ*M, Life Technologies) were diluted into DPBS and cells stained for 15 min at 24 °C, followed by washing 3 times in DPBS. Samples were mounted with antifade reagent (ProLong Gold, Life Technologies), dried overnight and imaged using a Zeiss Axiovert 40 CFL microscope and monochrome digital camera. Images were obtained using Zeiss Zen image acquisition software, saved as high-resolution, 8-bit TIFFs, and pseudocolored using ImageJ software.

Enzymatic staining of GLB1, FUCA1/2, HEXA/B, and MAN1/2 was performed with indolyl-based chromogenic enzyme substrates *X-Gal* (Gold Biotechnology, St. Louis MO, USA), *X-Fuca, X-Hex, and X-Man* (each from Santa Cruz Biotechnology, Dallas, TX, USA). Cells were grown in 6-well culture plates, treated with 2 *μ*M etoposide for 5 days to induce senescence, washed, fixed in 4% paraformaldehyde+0.25% glutaraldehyde for 4 min at 24 °C and washed twice in DPBS. Enzyme substrate stocks were prepared at 40 mg/ml in dimethylformamide (Sigma-Aldrich) and diluted to 1 mg/ml final in a staining buffer consisting of 5 mM K_3_Fe(CN)_6_, 5 mM K_4_Fe(CN)_6_•3H_2_O, and 2 mM MgCl_2_ in DPBS, adjusted to pH 4.0. Cells were stained for 18 h at 37 °C, washed once in DPBS, and then imaged under DPBS. Images were obtained using PlasDIC settings on an Axiovert 40 CFL microscope (Carl Zeiss Microscopy, Thornwood, NY, USA). ImageJ software was used to adjust background uniformly across images.

For LD staining, senescent or proliferating cells grown in culture dishes were fixed in 4% paraformaldehyde for 10 min at 24 °C and washed twice with DPBS. A solution of 0.25% Oil Red O (Sigma-Aldrich) in 60% isopropanol was prepared, filtered, and added to cells for 18 h at 24 °C. Cells were washed three times with dH_2_O and imaged using using PlasDIC settings on the Axiovert 40 CFL microscope. 20 images per sample were recorded and LDs per cell were counted manually using the ImageJ multipoint tool after background correction. Statistical analysis (unpaired *t-*test) was performed using GraphPad software.

To detect lipid uptake, NBD-C_6_-sphingomyelin (Setareh Biotech, Eugene, OR, USA), BODIPY-C_5_-ceramide (Setareh), BODIPY-C_11_ fatty acid probe (Life Technologies), NBD-C_6_-HPC phosphatidylcholine probe (Setareh), or NBD-cholesterol (Setareh) were each applied at 10 *μ*M to unfixed, live cells for 30 min at 37 °C. The cell samples were washed once with DPBS and then imaged using a Zeiss Axiovert 40 CFL epifluorescent microscope at ×20 magnification, using FITC filter set for each probe except for rhodamine to detect BODIPY-C_11_.

### Flow cytometric assays

For flow cytometric SA-*β*-Gal assays, trypsin-EDTA (Life Technologies) was used to detach adherent TIS cell monolayers or untreated controls for 5 min at 37 °C, immediately followed by neutralization of trypsin using an equal volume of complete cell culture media. Cells were then counted, aliquoted at 1×10^6^ cells per sample, and pelleted by centrifugation for 5 min at 1200×*g*. Cell pellets were resuspended in 1 ml of sterile DMEM-HI culture media (Life Technologies) without supplements or FBS. Bafilomycin A1 (1 *μ*M; Research Products International) was added and cells incubated 30 min at 37 °C without CO_2_ to alkalinize lysosomal pH. DDAOG (Life Technologies) was added at 10 *μ*g/ml from a 5 mg/ml stock solution in DMSO. Cells were incubated for 60 min at 37 °C without CO_2_, pelleted by centrifugation for 5 min at 1200×*g*, washed 1x in 1 ml of ice-cold 1% BSA-DPBS, pelleted by centrifugation, resuspended in 300 *μ*l cell viability stain (1 *μ*M Calcein Violet 450 AM (CV450, eBioscience, San Diego, CA, USA) in DMEM-HI without supplements or FBS), incubated 15 min on ice for and analyzed by flow cytometry (Fortessa, BD, San Jose CA, USA) with 10 000 cellular events analyzed per sample, detecting DDAOG with 640 nm excitation and 670 nm emission, and CV450 with 405 nm excitation and 450 nm emission.

For flow cytometric analysis of lipid uptake with SA-*β*-Gal staining, cells were harvested using 0.25% trypsin-EDTA for 5 min at 37 °C, resuspended in DMEM-HI culture medium without supplements or FBS, treated with fluorescent lipids as above, and then DDAOG (10 *μ*g/ml) was added to cell samples for 60 min at 37 °C. Samples were washed once with ice-cold 1% BSA-DPBS and resuspended in viability stain for 15 min on ice prior to flow cytometric analysis. Most lipids were detected using a 488 nm excitation laser with 525 nm emission detector, except for C_11_-BODIPY which was measured using a 561 nm excitation laser and 610 nm emission detector.

For assay of senescence in cells cultured with extracellular lipids, culture medium was prepared using 1x DMEM-HI (Life Technoloties), 10% delipidized FBS (Gemini Bioproducts), 4 mM l-glutamine (Gemini Bioproducts), and 1x pen/strep (Life Technologies). B16-F10 cells were plated at 5×10^3^ cells per cm^2^ and cultured in delipidized media for 24 h prior to addition of extracellular lipids as follows: l-α-phosphatidylcholine, 0.1 mM; cardiolipin, 0.5 mg/ml; sphingomyelin, 0.5 mg/ml; low-density lipoprotein, 0.1 mg/ml; cholesterol, 0.2 mg/ml; C_2_-ceramide (*N*-acetyl-D-sphingosine), 20 *μ*M; and C_2_-C_10_ triglyceride mix, 0.2 mg/ml. Lipids were dissolved in DMSO for at least 1 h at 37 °C except cardiolipin, which was provided in ethanol solution, and cholesterol, obtained in a water-soluble form. After 72 h, cultures were washed 2x with DPBS analyzed by flow cytometric SA-*β*-Gal assay.

For flow cytometric staining of cell surface proteins, cells were harvested by trypsinization, washed once in DPBS, counted, and resuspended at 1×10^6^ cells/ml in a blocking buffer of 5% normal goat serum (Jackson Immunological Research, West Grove, PA, USA) in 1% BSA-DPBS for 20 min on ice. Anti-LRP1 (Abcam), anti-PLD3 (ProteinTech Group, Rosemont, IL, USA), anti-LGALS3 (phycoerythrin-labeled conjugate, Novus Biologicals, Littleton, CO, USA) and anti-LGALS9 (Alexa Fluor 594-labeled conjugate, Novus) were applied to cells in 100 *μ*l blocking buffer for 30 min at 4 °C. For fluorophore-labeled primary antibodies, cells were washed once in 1 ml of ice-cold 1% BSA-DPBS and then incubated for 15 min with a viability stain for 15 min on ice immediately prior to analysis. For unlabeled primary antibodies, cells were washed once in 1 ml of ice-cold 1% BSA-DPBS and incubated for 30 min with fluorescent secondary antibody in 500 *μ*l of 1% BSA-DPBS+5% normal goat serum. Anti-LRP1 was detected with anti-rabbit labeled with Alexa Fluor 647 (Jackson); anti-PLD3 was detected by anti-rabbit labeled with DyLight 594 (Life Technologies). Samples were then washed once in 1 ml of ice-cold 1% BSA-DPBS, and incubated 15 min with viability stain for 15 min on ice immediately prior to analysis.

For aldehyde detection using the Alexa Fluor 568 Hydrazide aldehyde-reactive probe (Life Technologies), TIS cells induced as described above were loaded with the probe via pinocytosis. Briefly, pinocytic cell loading reagent (Influx, Life Technologies) was prepared according to manufacturers’ instructions by heating reagent vials for 5 min to melt the PEG cap using an 80 °C water bath, adding 4.7 ml of prewarmed culture medium (without serum) per vial, mixing vigorously at 37 °C for 30 min to dissolve sucrose crystals, and then adding FBS (5% v/v) and sterile HEPES (10 mM). Alexa Fluor 568 Hydrazide was then dissolved in prepared pinocytic loading reagent at 10 *μ*M, and this solution was applied to label live cells for 30 min at 37 °C (5% CO_2_). Pinocytic lysis medium was prepared as a 6 : 4 solution of sterile dH_2_O:DMEM-HI (no serum). Following pinocytic hydrazide probe labeling, loading medium was completely removed and replaced with pinocytic lysis medium for exactly 1 min, followed by removal of lysis medium and replacement with complete culture medium for 30 min at 37 °C (5% CO_2_) to allow cell recovery. Cells were then harvested via mechanical dissociation (cell scraper), pelleted by centrifugation at 1200×*g*, washed 2x in ice-cold 1% BSA-DPBS to remove any traces of unbound probe, resuspended in 300 *μ*l of DMEM-HI (no serum), and placed on ice for transport to flow cytometer for immediate analysis. Alexa Fluor 568 Hydrazide signal was resolved using a 561 nm excitation laser and a 610 nm emission detector, according to manufacturer recommendation; lipofuscin autofluorescence was co-analyzed for each sample using a 488 nm excitation laser and 525 nm emission detector. 10 000 cells per sample were analyzed. Dead cells and debris were eliminated from analysis by conventional light scatter gating. Establishment of a gate to identify aldehyde-high, lipofuscin-high cells was conducted as shown in Supplementary Figure 6.

Aldehyde dehydrogenase activity in living cells was assayed using the AldeRed ALDH detection assay kit (EMD Millipore, Burlington, MA, USA) according to manufacturer's instructions. In brief, AldeRed-588 reagent was dissolved in DMSO with 2 N HCl for 15 min at 24 °C (room temperature). Verapamil was added to the AldeRed assay buffer to prevent probe efflux from cells. When the AldeRed-588 probe was dissolved, assay buffer was added and this solution was used to label cells. TIS cells were prepared as described above, harvested via trypsinization, counted, and resuspended at 1×10^6^ in 1 ml of assay buffer containing AldeRed-588 probe for 60 min at 37 °C. After staining, cells were pelleted by centrifugation for 5 min at 1200×g, resuspended in 500 *μ*l of ice-cold assay buffer, and kept on ice for immediate flow cytometric analysis. AldeRed-588 signal was resolved using a 561 nm excitation laser with a 610 nm emission detector, as suggested by probe manufacturer.

For all flow cytometry assays, cell samples were analyzed immediately following the staining procedures using a BD Fortessa flow cytometer. At least 10 000 cells were analyzed per sample. Data acquisition parameters were set using instrument calibration particles (SpheroTech) and FACSDiva software (BD). Raw data (.fcs) was exported to FlowJo software for analysis. Dead cells and debris were excluded from populations prior to statistical analysis using gating strategies to identify intact and viable cells as shown in Supplementary Figures 5 and 6. Statistical significance tests for flow cytometry data were conducted using unpaired, two-tailed *t*-tests to generate *p*-values using raw fluorescence data from *n*=500 intact/viable cell events.

### Western blotting

Cells were pelleted following FACS and immediately lysed in ice-cold RIPA buffer consisting of 150 mM NaCl (Fisher Bioreagents, Asheville, NC, USA), 50 mM Tris base (Fisher Bioreagents), 1% Triton-X-100 (Sigma-Aldrich), 0.5% sodium deoxycholate (Sigma-Aldrich), 0.1% SDS (Sigma-Aldrich), adjusted to pH 8.0. Lysis was conducted for 30 min on ice with rapid vortexing every 10 min. Protein concentration of lysates was determined using a BCA assay kit (Pierce). Lysates were diluted into denaturing gel loading buffer containing 2% SDS (Sigma-Aldrich), 1% beta-mercaptoethanol (GE Healthcare Biosciences, Marlborough, MA, USA), 10% glycerol (Sigma-Aldrich), 12.5 mM EDTA, and 0.02% bromophenol blue (Sigma-Aldrich), pH 6.8. Samples were loaded onto 4–12% gradient Bis-Tris acrylamide gels (Life Technologies) at 10 *μ*g/lane, and electrophoresis was conducted for 50 min at 200 V constant using MOPS buffer (Life Technologies). Gels were transferred onto nitrocellulose membranes using a wet-transfer system (Life Technologies). Protein transfer was confirmed using reversible protein stain (Memcode, Life Technologies). Total protein staining is shown as the loading control in figures for each blot. Membranes were blocked for 30 min in 5% BSA-TBS and washed once in a TBS-T buffer consisting of TBS 1x (150 mM NaCl+50 mM Tris Base, pH 7.4)+0.1% Tween-20 (Sigma-Aldrich). Primary antibodies were then diluted in 1% BSA-DPBS at manufacturer recommended titration (typically 1 : 200–1 : 1000 or ~0.2–1.0 *μ*g/ml), and incubated with membranes overnight at 4 °C with rotation. Primary antibodies included anti-GBA (Abcam); anti-SGPP1 (Santa Cruz Biotechnology); anti-SMPD1 (Santa Cruz Biotechnology), anti-ANXA6 (Santa Cruz Biotechnology); anti-CPT1A (ProteinTech Group); anti-DGAT1 (GeneTex, Irvine CA); anti-PEX1 (ProteinTech Group); anti-PLIN2 (Novus Biologicals); anti-CLN5 (Abcam), and anti-ALDH2 (Novus Biologicals). Membranes were then washed three times with TBS-T. HRP-conjugated secondary antibodies (anti-rabbit:HRP and anti-mouse:HRP, GE Healthcare, 1 : 10 000 dilution; anti-goat:HRP, EMD Millipore, 1 : 50 000 dilution) were diluted in 5% BSA-DPBS and added for 30 min at 4 °C to detect the primary antibodies, followed by washing three times in TBS-T. Chemiluminescent HRP substrate (ECL Pico or Femto, Life Technologies) was added and membranes exposed to autoradiography film. In some cases, blotting membranes were treated with ‘Restore’ blot stripping buffer (Life Technologies), reblocked, and then reprobed.

## Additional information

**Publisher’s note:** Springer Nature remains neutral with regard to jurisdictional claims in published maps and institutional affiliations.
