# Supplementary material for: A signature of enhanced lipid metabolism, lipid peroxidation and aldehyde stress in therapy-induced senescence
Source: Cell Death Discov. 2017 Oct 30;3:17075–. doi: 10.1038/cddiscovery.2017.75 (PMC5661608; doi:10.1038/cddiscovery.2017.75)
Supplement: Supplementary Information [file cddiscovery201775-s1.docx]

**Supplementary Tables and Figures**


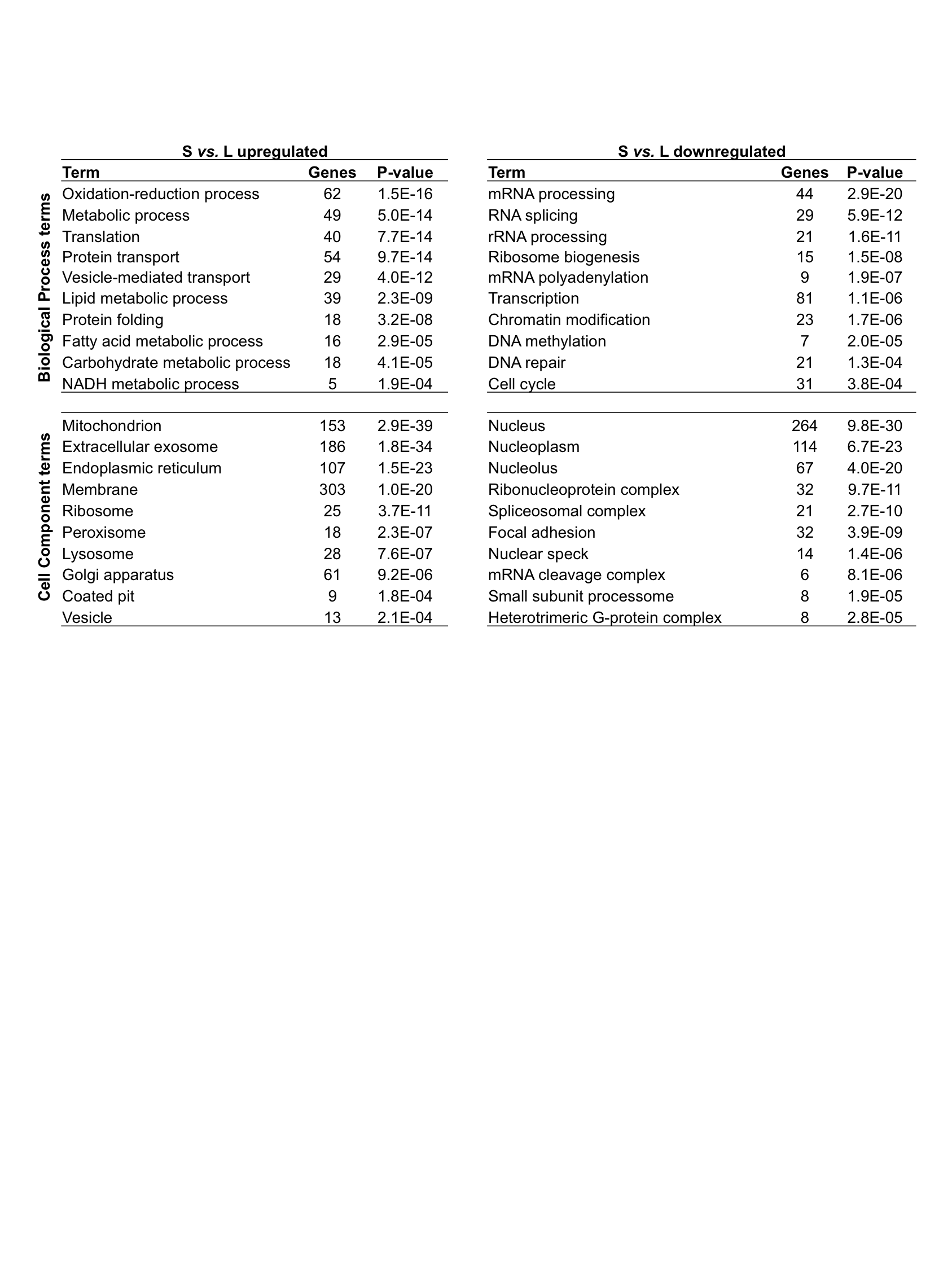


**Supplementary Table 1. Proteomics analysis of etoposide-treated cells expressing**

**SA-β-Gal.** Gene Ontology (GO) terms that differentiate SA-β-Gal^HI^ (S) and SA-β-Gal^LO^ (L) senescent cells. Upregulated categories for S *vs*. L include mostly cytoplasmic processes, including redox process and several GO terms related to lipid metabolism. Downregulated categories point to ribosomal and nuclear processes.


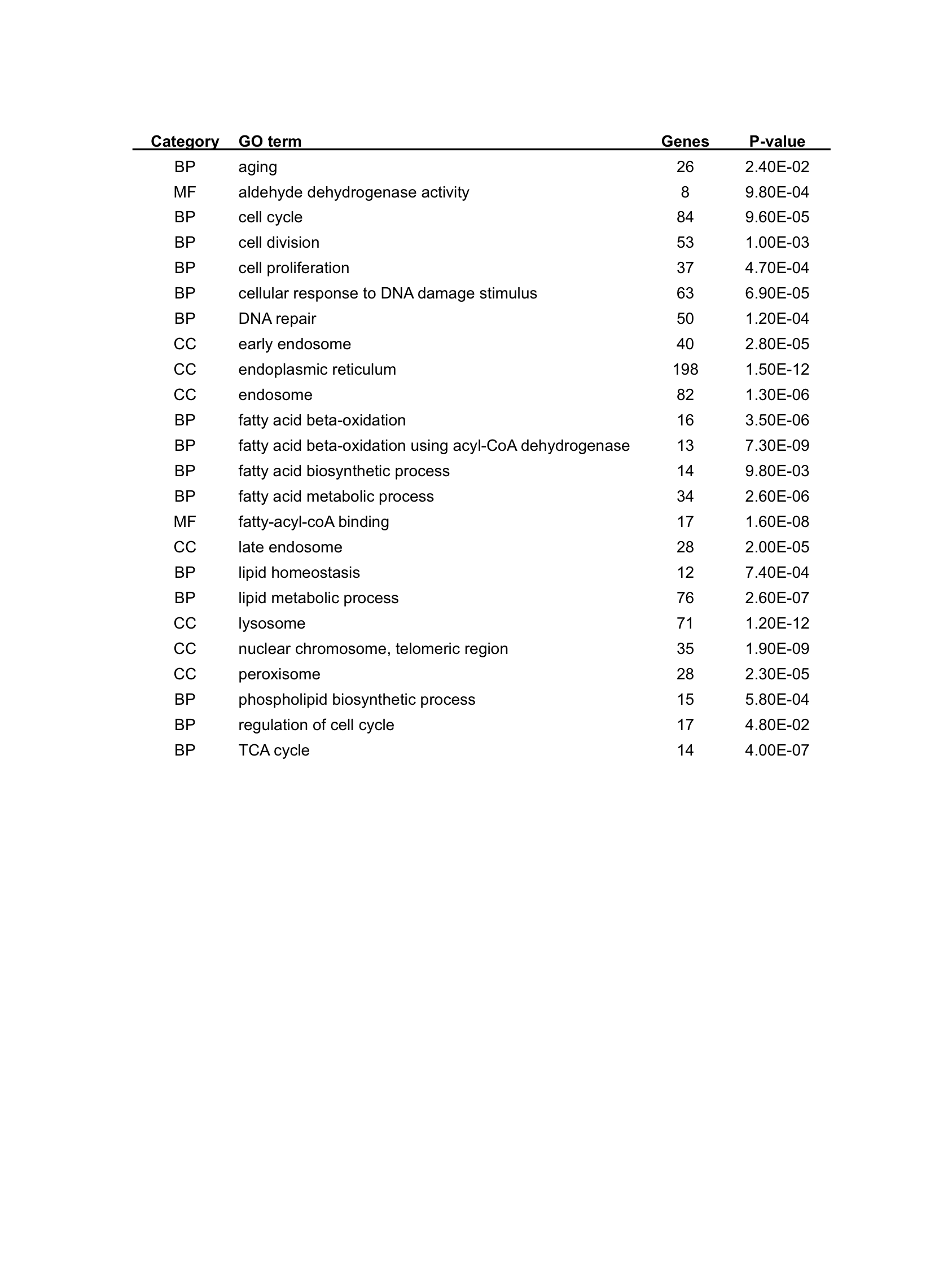


**Supplementary Table 2. Enriched GO terms related to cellular lipid regulation for senescent *vs*. proliferating cells.** GO analysis based on proteins ≥ 1.5-fold upregulated in senescent SA-β-Gal^HI^ (S) *vs.* proliferating (P) cells. Categories are BP = Biological Process; CC = Cell Component; MF = Molecular Function.


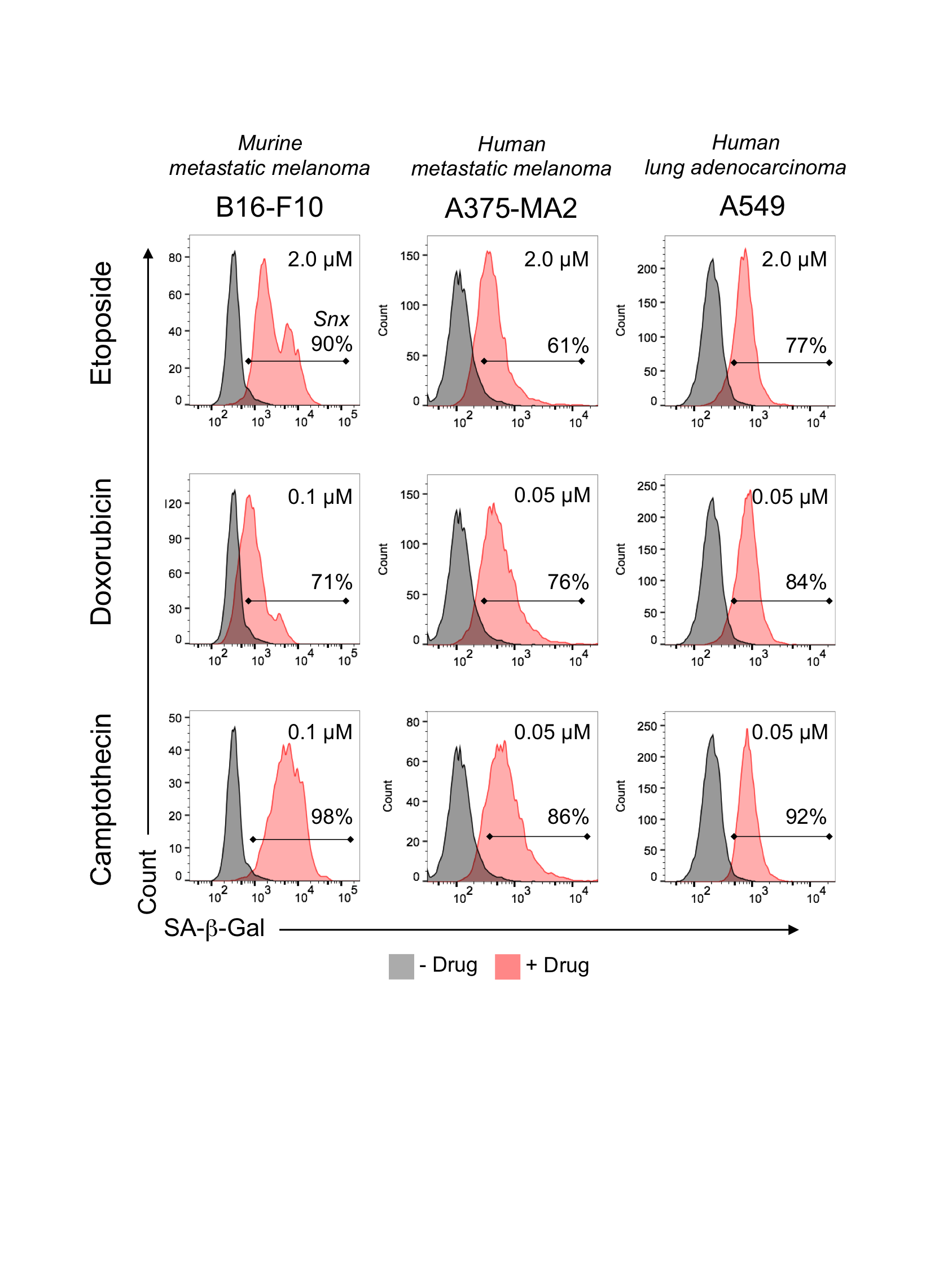


**Supplementary Figure 1. Detection of therapy-induced senescence in murine and human cell lines by flow cytometric SA-β-Gal assay.** Murine (B16-F10) and human (A375-MA2) melanoma and human lung adenocarcinoma (A549) cell lines were treated with etoposide, doxorubicin, or camptothecin for 96 h at concentrations indicated and assayed for senescence using the DDAOG SA-β-Gal assay. Each cell line and treatment condition exhibits onset of TIS at percentages indicated on histogram plots.


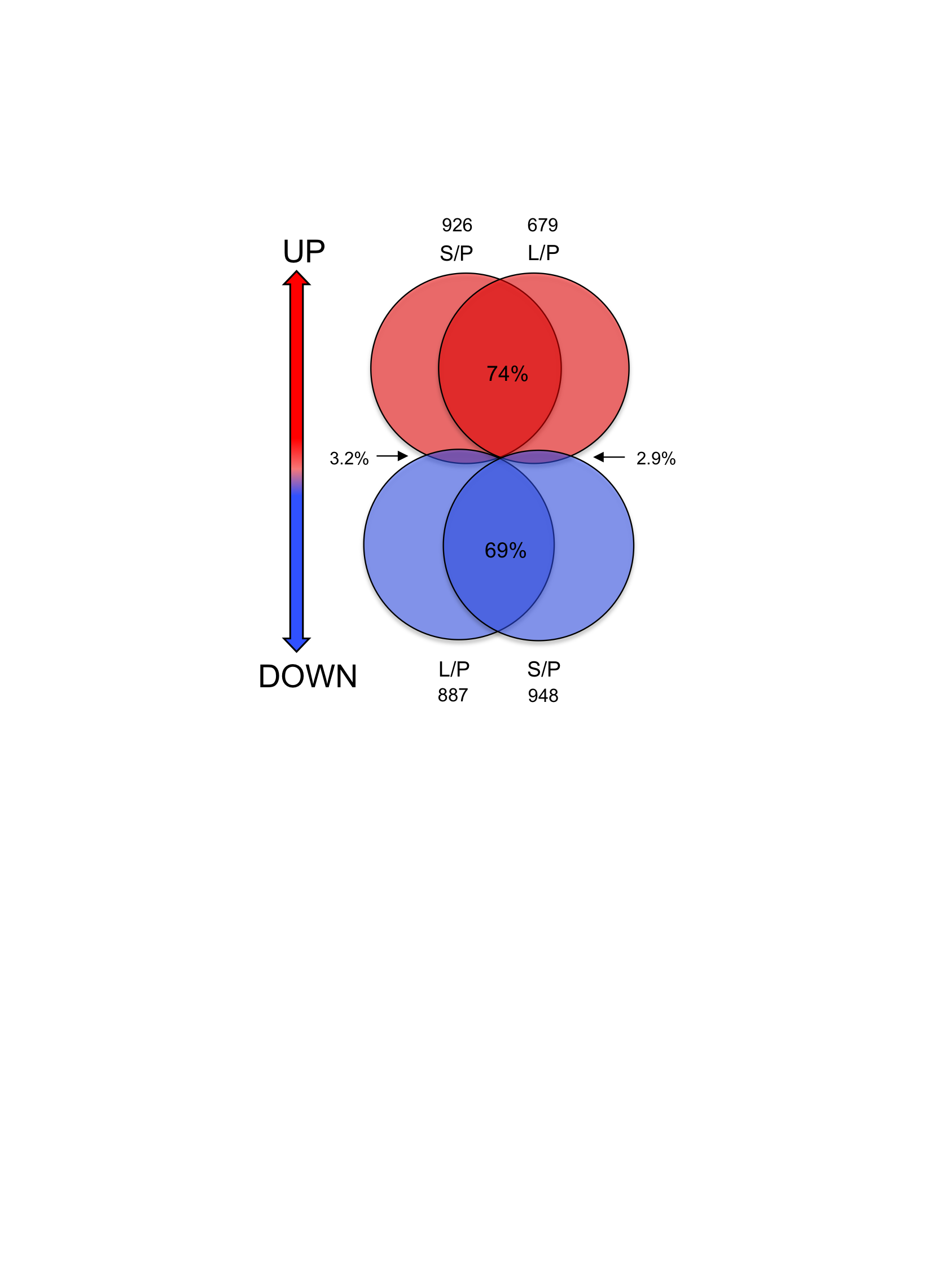


**Supplementary Figure 2. Proteomics analysis of etoposide-treated cells expressing SA-β-Gal.** Venn diagram showing trends in upregulated *vs*. downregulated proteins for SA-β-Gal^HI^ (S) and SA-β-Gal^LO^ (L) etoposide-treated cells, as compared to proliferating cells (P). 74% of upregulated proteins and 69% of downregulated proteins were shared between S and L samples.

**
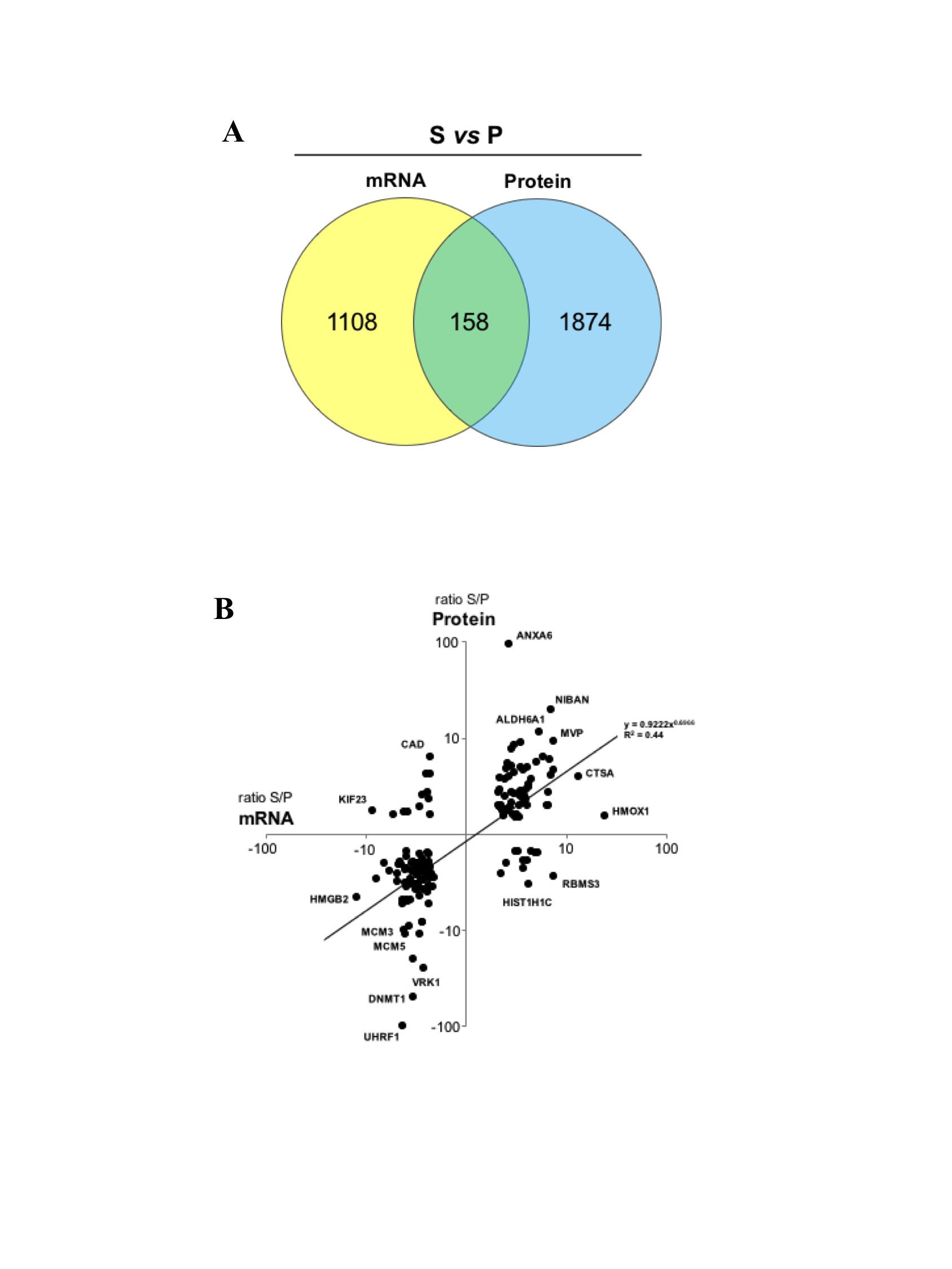
Supplementary Figure 3. Transcriptomics data analysis summary. (A)** Venn diagram showing overlap of genes identified to show significant change in both transcriptomics and proteomics analysis. Analysis of sorted SA-β-Gal^HI^ senescent (S) vs. proliferating (P) cells is shown. Significant fold change in at least two replicates was required. Approximately 10% of total identified genes were shared between data sets. **(B)** Quad plot showing S/P ratio data in mRNA transcriptomic analysis vs proteomics data. A moderate degree of correlation was seen. Shared upregulated genes include aldehyde dehydrogenase ALDH6A1 and annexin ANXA6; shared downregulated genes include multiple nuclear factors, such as checkpoint genes MCM3/5, chromatin-associated DNA repair protein HMGB2, and epigenetic factors DNMT1 and UHRF1.


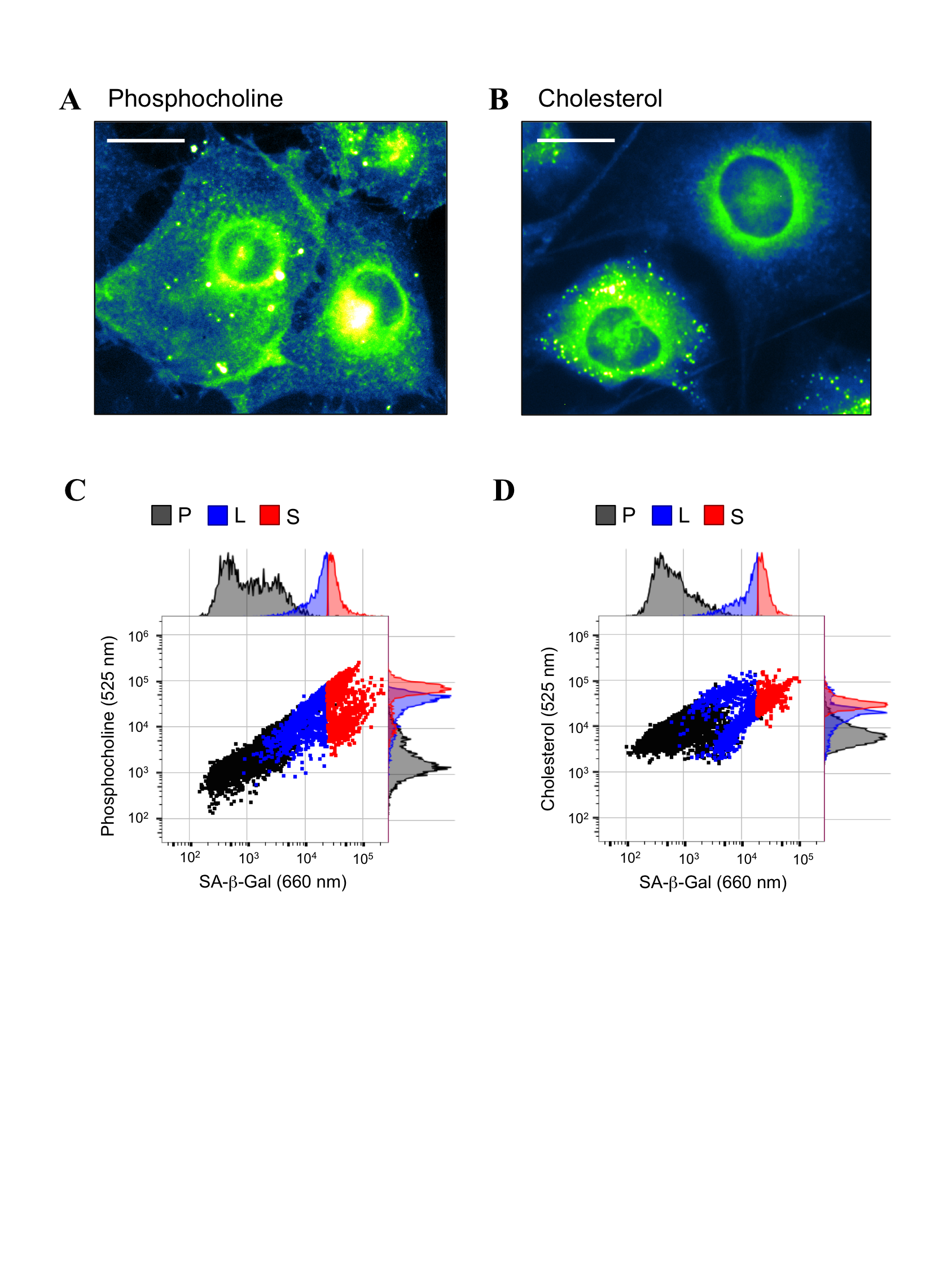


**Supplementary Figure 4. Uptake of fluorescent phosphocholine and cholesterol by senescent cells. (A, B)** Imaging of etoposide-induced senescent cells showing uptake of fluorescent phosphocholine (**A**) or cholesterol (**B**) probes after 30 min incubation.

**(C, D)** Flow cytometric analysis of lipid uptake *vs.* SA-β-Gal for phosphocholine **(C)** or cholesterol **(D)**.


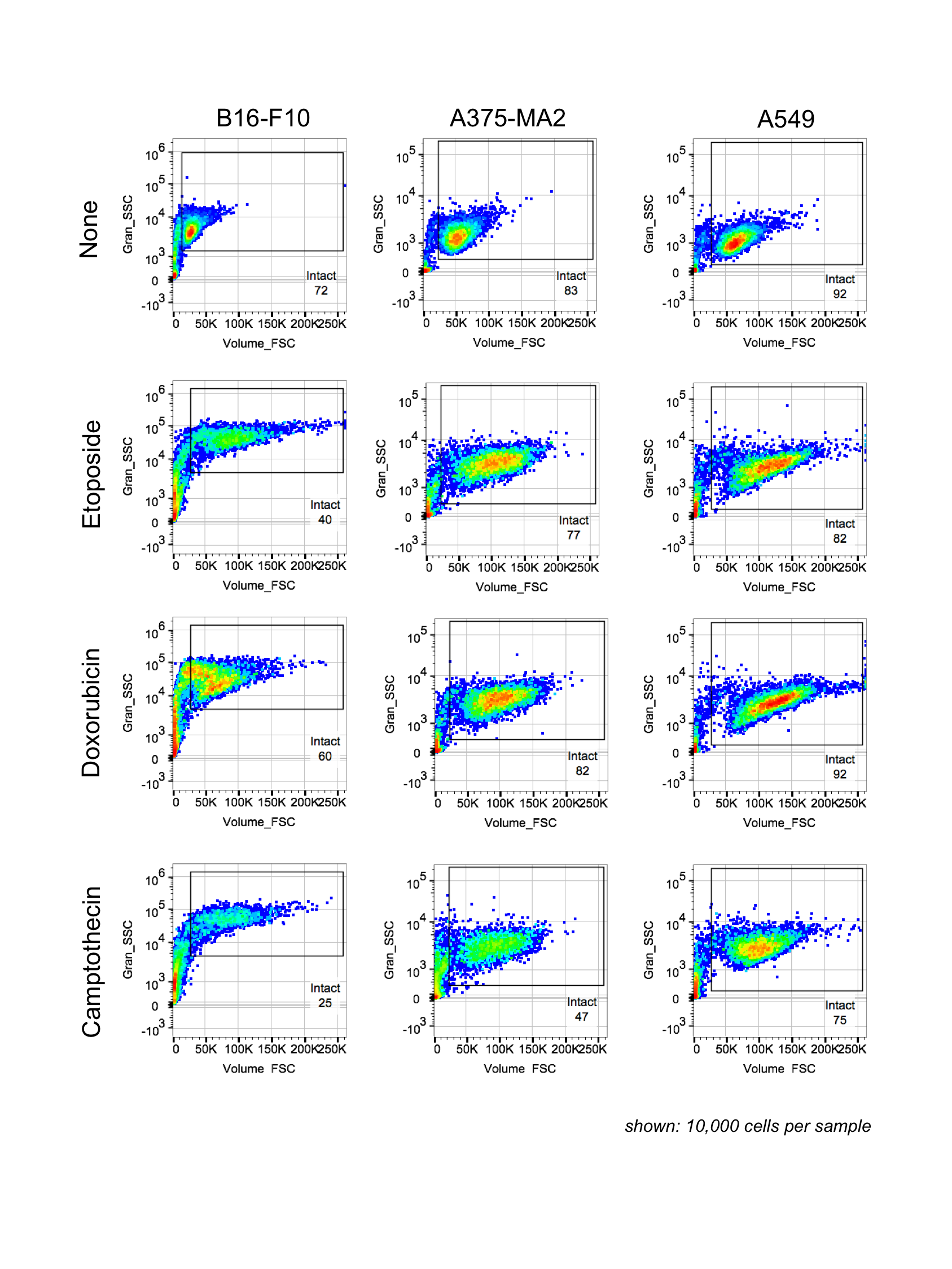


**Supplementary Figure 5. Light scatter gating to identify intact cells.** Intact cells were separated from dead cell debris by a conventional light scatter gating approach using forward scatter (FSC, cell volume) *vs*. side scatter (SSC, cellular granularity) plots. Intact cells and, particularly, larger senescent cells display high FSC and SSC compared to debris. Gated intact cells were further analyzed for viability (**Supplementary Fig. 6**).


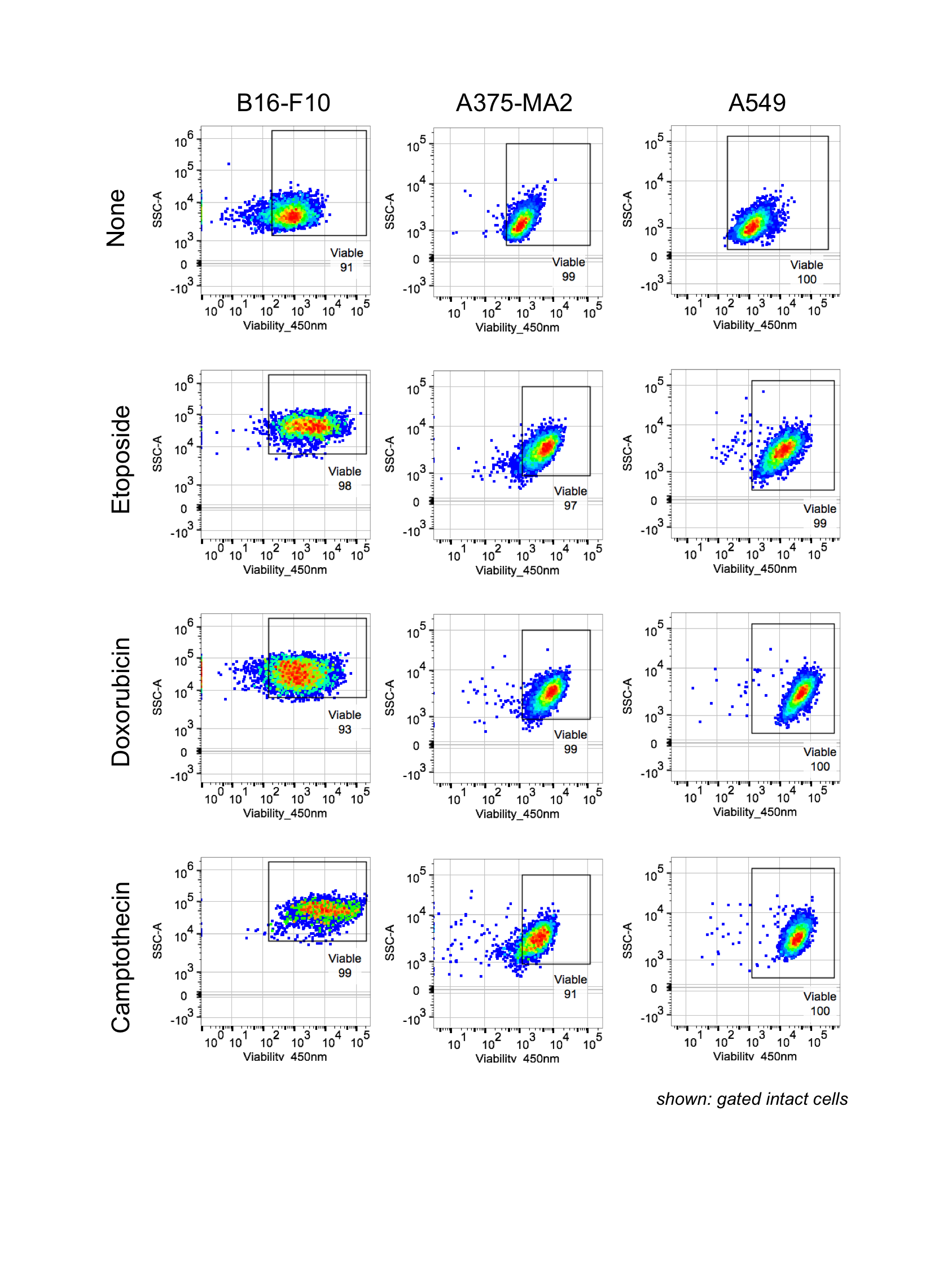


**Supplementary Figure 6. Viability stain gating to identify viable cells.** Viable intact cells were separated from dying/dead intact cells using Calcein Violet AM 450 as a cellular viability probe, measured at 450 nm emission. Gated viable cells were then further analyzed for senescence (e.g. **Fig. 1A** and **Supplementary Fig. 1**) and/or ROS (**Supplementary Fig. 7**).


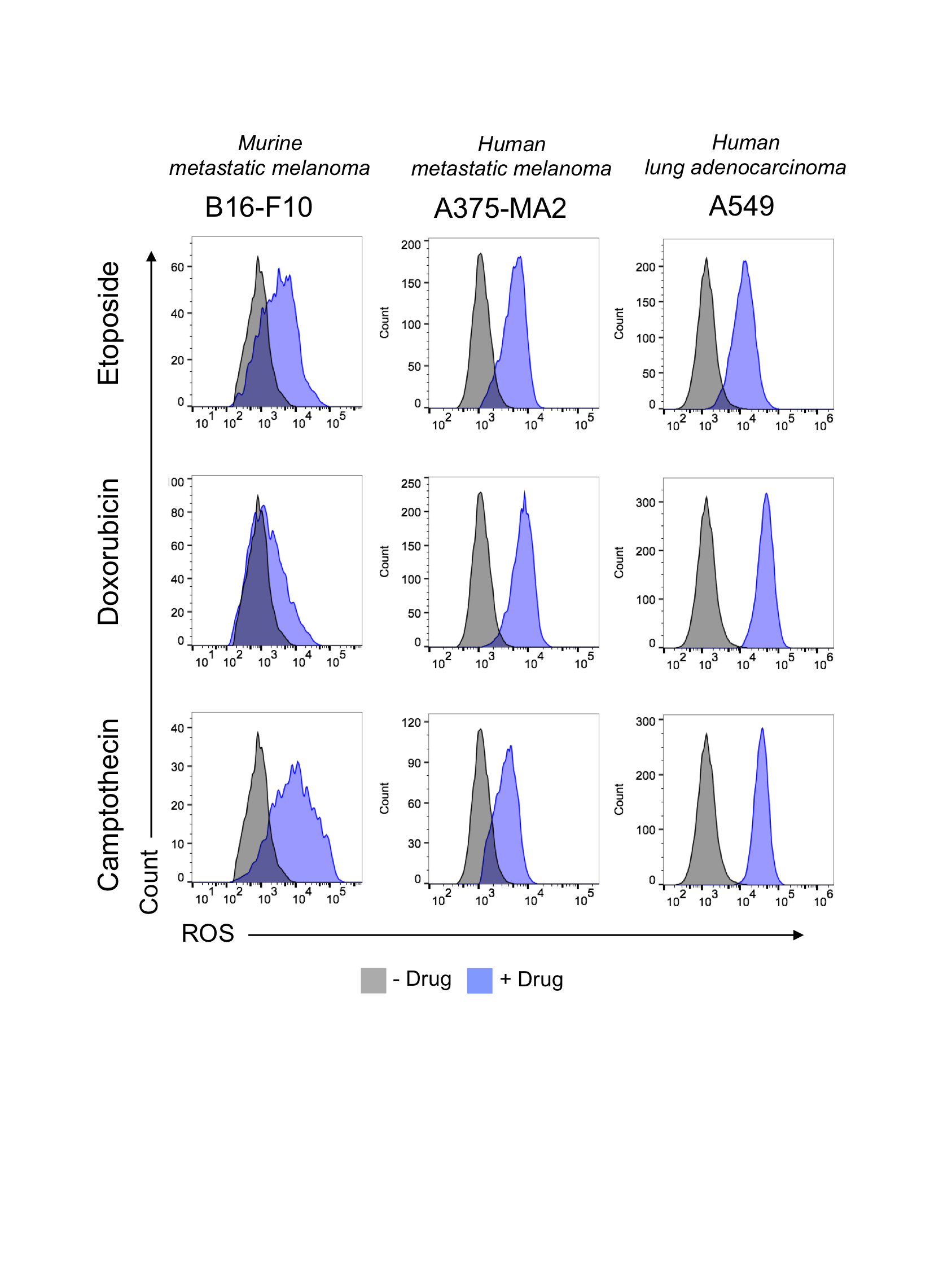


**Supplementary Figure 7. Reactive oxygen species (ROS) levels during TIS.** Reactive oxygen species were assayed using the Calcein Violet AM 450 probe, which is a redox-sensitive probe typically used for cellular viability assays. Each of three cell lines (murine and human metastatic melanoma and human lung adenocarcinoma) were assayed for ROS following 96 h of treatment with chemotherapeutics etoposide, doxorubicin, or camptothecin at doses sufficient to induce senescence.
